# A Bayesian hierarchical hidden Markov model for clustering and gene selection: Application to kidney cancer gene expression data

**DOI:** 10.1002/bimj.202300173

**Published:** 2024-06

**Authors:** Thierry Chekouo, Himadri Mukherjee

**Affiliations:** 1 Division of Biostatistics and Health Data Science, School of Public Health, University of Minnesota, Minnesota, USA; 2 Department of Mathematics and Statistics, University of Minnesota Duluth, Minnesota, USA

**Keywords:** Bayesian hidden Markov model, biclustering, biological prior knowledge, kidney cancer

## Abstract

We introduce a Bayesian approach for biclustering that accounts for the prior functional dependence between genes using hidden Markov models (HMMs). We utilize biological knowledge gathered from gene ontologies and the hidden Markov structure to capture the potential coexpression of neighboring genes. Our interpretable model-based clustering characterized each cluster of samples by three groups of features: overexpressed, underexpressed, and irrelevant features. The proposed methods have been implemented in an R package and are used to analyze both the simulated data and The Cancer Genome Atlas kidney cancer data.

## 1 | INTRODUCTION

In pathology and cancer research, where conditions of a study refer to different tumor samples from which different messenger RNA (mRNA) samples are taken, it is reasonable to infer that tumor samples of the same pathological type should have similar expression levels for each of those genes that play a responsible role for the pathology. From a molecular biology perspective, genes that share similar expression profiles are of biological interest since they either have a similar function or are involved in a common biological process often controlled by the same transcriptional regulatory program, or are members of the same pathway or protein complexes, and hence functionally similar (Wikipedia Contributors, 2018). This forms the basic motivation for various clustering algorithms on microarray data, where a goal is to select genes that are coexpressed under all the experimental conditions (e.g., different environmental samples, different individuals or tissues, or different time points). In other words, all genes in a given cluster must show similar coregulation patterns across all experimental conditions. Therefore, clustering algorithms are a sensible choice in situations where experiments are conducted under a limited number of homogeneous experimental conditions.

Now, with the advancement of sequence technologies and availability of publicly available repositories of omics data, such as TCGA (The Cancer Genome Atlas)(The Cancer Genome Atlas Research Network et al., 2013), we need to perform a similar analysis but with data from a heterogeneous compendium. In this case, the assumption of applying conventional clustering techniques may not hold as genes that share similar expression patterns only exhibit coexpression under a subset of conditions. Biclustering algorithms have the advantage of discovering genes that are coexpressed in a subset of conditions (instead of all), compared with conventional clustering methods. Moreover, since gene expression levels are measured under a large number of heterogeneous conditions, biclustering suits the need for the type of system-level analysis we need for discovering transcriptional modules, which provide essential clues for revealing genetic networks.

Although existing biclustering algorithms originate with the same goal in mind, to cluster both genes and samples at the same time, they differ from each other by the approach they use on the problems they try to solve. Among early work on biclustering methods, algorithms were designed to divide the data into checkerboard units of patterns-clustering algorithms were applied iteratively to both the genes and samples of the data (Alon et al., 1999). There are also algorithms particularly designed keeping the biclustering goals in mind.

Our model approach belongs to the class of biclustering algorithms where we question conventional clustering algorithms by the idea that genes that share functional similarities do not have to be coexpressed over all the samples in the data set. This idea has been used to develop clustering algorithms that are used to cluster genes that share similar expression patterns over a subset of samples, and similarly, it has been used to cluster tumor samples that share similar gene expression levels over a subset of genes. This type of algorithm where the problem has been broken down into two separate orientations-biclustering genes and samples, was first pioneered by Cheng and Church (2000). Multiple biclustering algorithms have been proposed in the literature (Chekouo & Murua, 2015; Chekouo et al., 2015; Cho et al., 2004; Getz et al., 2000; Gu & Liu, 2008; Lee et al., 2010; Sill et al., 2011; Turner et al., 2005; Xue et al., 2014; Zhang, 2010). For instance, Tanay et al. (2002) discretize the gene expression values into three levels-upregulated, downregulated, and inactive and represents the discrete matrix as a bipartite graph. It then uses a heuristic approach to find bicliques in the graph, which correspond to biclusters in the matrix. The plaid model of Lazzeroni and Owen (2002) is also one of the popular biclustering methods. The spectral biclustering method (Kluger et al., 2003) uses singular value decomposition to solve the biclustering problem.

Most of these algorithms are not able to incorporate prior knowledge in their applications. In contrast, Bayesian models provide our method with a systematic base for the integration of prior knowledge and information from other data sources. The work of Chekouo et al. (2015) is an example of a Bayesian biclustering method that integrates other data sources.

Our proposed biclustering method in this manuscript not only circumvents these shortcomings but it also encompasses much of the rich structure in the genomic expression data. Moreover, the proposed method identifies "predefined" biclusters that are biologically meaningful and interpretable, that is, characterized by genes/features that are overexpressed and underexpressed. Since we use a single coherent probabilistic framework, the model is extendable and can incorporate other functional information, such as experiment type, putative binding sites, and so forth. Since genes can be sorted based on their annotation congruence (Christophe & Nives, 2017; Thomas et al., 2012), our proposed model takes advantage of this potential coexpression between neighboring genes by imposing first-order Markovian assumption on hidden gene states. Typically, as with other biclustering models, ours is an incomplete data problem and an unknown model structure. We will treat the hidden variables (gene labels and cluster labels) and the model parameters as random variables, and use Gibbs sampling to estimate their joint distributions. To sort genes based on functional similarity, we use the *mgeneSim* method in the Bioconductor package GoSemSim (Li et al., 2010) to compute the semantic similarity among multiple gene products.

The manuscript is organized as follows. In Section 2, we describe our Bayesian model-based (bi)clustering using the hidden Markov model (HMM). In Section 3, we present a simulated data study. Section 4 describes the application to TCGA kidney cancer data. We conclude the manuscript in Section 5.

## 2 | A BAYESIAN BICLUSTERING APPROACH USING HMM

In the context of model-based clustering for high-dimensional features, the feature space is usually divided into two nonoverlapping sets (Chekouo & Murua, 2018; Fop & Murphy, 2018); variables or features which are relevant for clustering and whose distribution directly depends on the group membership variable, and irrelevant variables which may be further divided into either redundant variables (i.e., contain similar information as relevant variables as they are correlated with the relevant ones but from the point of distributional representation are conditionally independent of the grouping variable Z given the relevant variable), or uninformative variables (i.e., correspond to noise and their distribution is completely independent of the group structure) (Maugis et al., 2009). In our modeling context, we will select variables within each cluster to be either relevant (overexpressed or underexpressed in most of the conditions/samples) or irrelevant (not significantly expressed but might be correlated to relevant ones).

**FIGURE 1:**
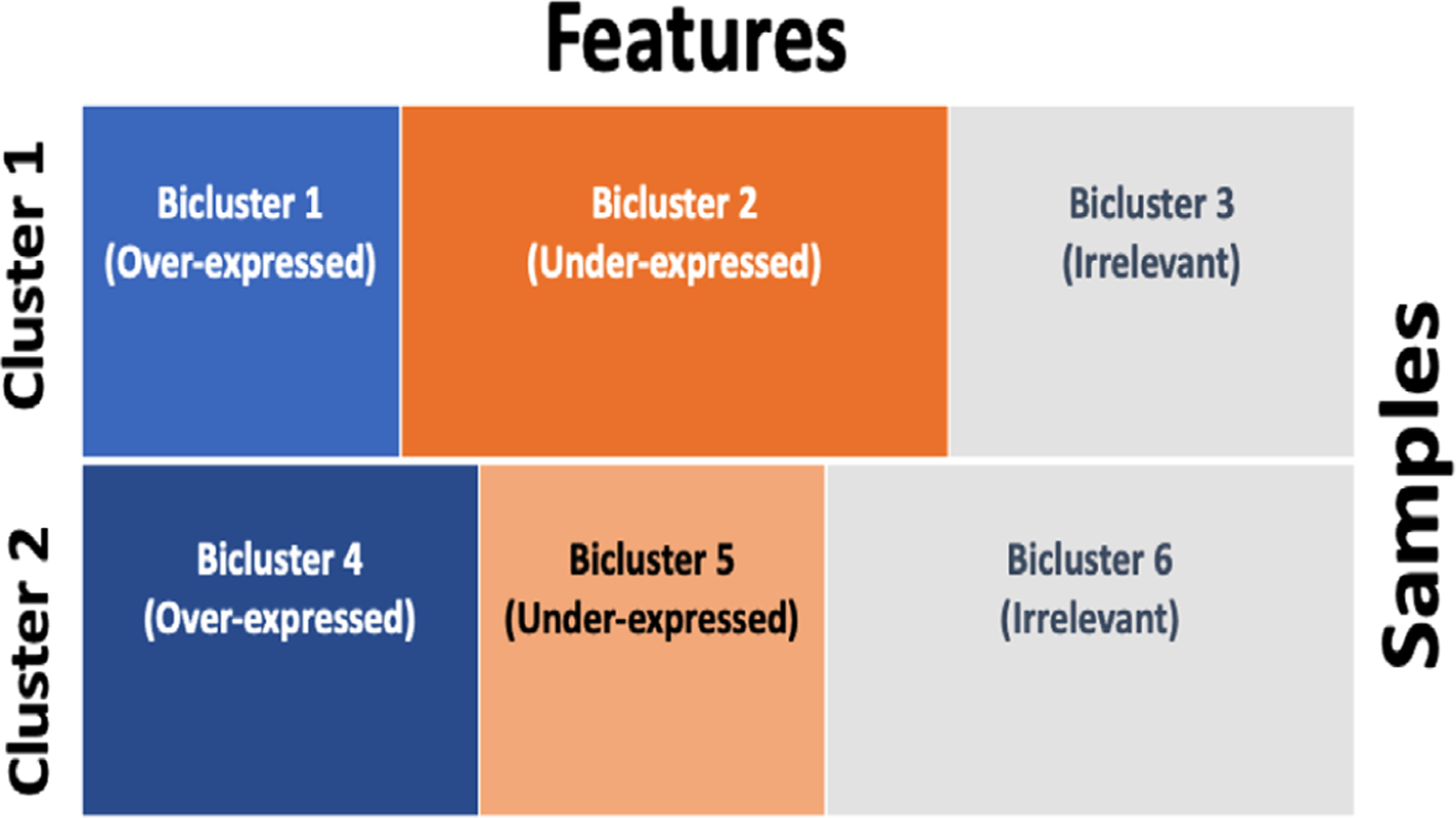
An example of a biclustering structure captured by our approach. In this example, we have K=2 clusters, and K×3=6 biclusters.

In our approach, we assume a dependence structure between features via prior distributions of the feature indicator variables $\left(\rho \right)$. In fact, the feature selection process within each cluster uses first-order Markov assumption between feature variables. For each sample/subject cluster, we model gene expression across samples using an HMM with three states/groups: overexpressed genes (i.e., state 1 ), underexpressed genes (i.e., state 2 ) and not actively expressed genes (i.e, state 3) (Cui et al., 2015). Since genes will be sorted/ordered based on their functional similarity, the HMM can take advantage of potential coexpression by borrowing information across closer genes in their ordering structure. Hence, our biclustering results would not depend on the arbitrary order in which the p features are presented but, instead, they will depend on a prior ordering structure of features defined using biological knowledge (e.g., Gene Ontology [GO]). With this assumption, genes/features that are closer in the ordering structure will be more likely to belong to the same feature state. Within each sample cluster, we have three groups of features (i.e., three states), which will correspond to a total of 3×K biclusters where K is the number of sample clusters. Figure 1 illustrates an example of a biclustering structure captured by our approach with K=2 clusters.

### 2.1 | The approach

For each cluster k=1,…,K, the latent feature indicator vector $\rho_{j,k}$, will define feature expression with three possible states; $\rho_{j,k}$ is 1,2, and 3 according to whether column expression value for gene j in cluster k is viewed as overexpressed, underexpressed, or not actively expressed (i.e., irrelevant), for every feature $j\in \left\{1,2,3,\ldots ,p\right\}$, respectively (see Figure 1 for example). Let $z_{i}$ be the cluster indicator variable for individual/sample i, where $z_{i}=k$ if individual i belongs to cluster $k\in \left\{1,2,\ldots ,K\right\}$. Let $y_{ij}$ be the matrix of values (e.g., gene expression) of feature j expressed under sample i. We assume that given the cluster label k=1,…,K, and state $l=1,2,3,y_{ij}$ follows independent (with respect to i and j) distributions defined as

(1) $$y_{ij}=\mu_{jkl}+\epsilon_{ijkl},\;\epsilon_{ijkl}∼𝓝\text{ormal}\left(0,\sigma_{jkl}^{2}\right),$$

where $\sigma_{jkl}^{2}$ is the error variance. We define a hierarchical model by defining further prior distributions on $\mu_{jkl}$ and $\sigma_{jkl}^{2}$. In fact, we assume that

(2) $$\mu_{jkl}∼\left\{\begin{array}{cc} 𝓝\text{ormal}\;\left(\mu_{k1},\sigma_{k1}^{2}\right), & \text{if}\;\mu_{k1}>0\;\text{and}\;\rho_{j,k}=1\;\text{(relevant}\;\text{and}\;\text{overexpressed),}\\ 𝓝\text{ormal}\;\left(\mu_{k2},\sigma_{k2}^{2}\right), & \text{if}\;\mu_{k2}<0\;\text{and}\;\rho_{j,k}=2\;\text{(relevant}\;\text{and}\;\text{underexpressed),}\;\\ 0, & \text{if}\;\rho_{j,k}=3\;\text{(irrelevant),}\; \end{array}\right.$$

where $\mu_{k1}>0$ and $\mu_{k2}<0$ are the parameter means of overexpressed and underexpressed features, respectively, $\sigma_{k1}^{2}$ and $\sigma_{k2}^{2}$ are their corresponding variances within cluster k. From Equation (2), we can see that each cluster k is characterized by three different means:$\mu_{k1}$, $\mu_{k2}$, and 0 that corresponds, respectively, to the mean averages of overexpressed, underexpressed, and no active features. Our model (1 and 2) identifies irrelevant features (i.e., redundant) within each cluster k (not across all cluster k) which is different from standard methods that perform simultaneously clustering and variable selection (Chekouo & Murua, 2018; Fop & Murphy, 2018; Maugis et al., 2009). However, in our model, redundant/irrelevant features across all clusters (i.e., $\rho_{j,k}=3$ for every k) can be considered unimportant features. Moreover, we also assume that $\sigma_{jk3}^{2}=\sigma_{j3}^{2}$ for every k, that is, independent of subject clustering.

In order to incorporate the prior ordering structure of our features into our model, we define a first-order Markov dependency between latent feature variables in cluster k, that is,

$$p\left(\rho_{j,k}|\rho_{k}^{\left(j-1\right)}\right)=p\left(\rho_{j,k}|\rho_{j-1,k}\right),\;j=2,3,\ldots ,p,$$

where $\rho_{k}^{\left(j-1\right)}=\left(\rho_{1,k},\rho_{2,k},\ldots ,\rho_{j-1,k}\right)$ denotes sequence history up to sequence point j−1, and $\rho_{j-1,k}$ denotes the state of feature j−1 sequence point within cluster k. $p\left(\rho_{j,k}|\rho_{j-1,k}\right)$ represents the transition probability from state $\rho_{j-1,k}$ to state $\rho_{j,k}$ within cluster k. Within cluster k, the observation $\left(y_{ij}\right)$ depends only on the latent state $\left(\rho_{j,k}\right)$. If $y_{i}^{\left(j-1\right)}=\left(y_{i1},y_{i2},\ldots ,y_{i,j-1}\right)$, then

$$p\left(y_{ij}|y_{i}^{\left(j-1\right)},\rho_{k}^{\left(j\right)},z_{i}=k\right)=p\left(y_{ij}|\rho_{j,k},z_{i}=k\right).$$

Hence, our complete HMM likelihood can be written as

(3) $$p\left(y,\rho ,z|\theta \right)=\prod_{i=1}^{n}p\left(\rho_{1,z_{i}}\right)\prod_{j=2}^{p}p\left(\rho_{j,z_{i}}|\rho_{j-1,z_{i}}\right)p\left(z_{i}\right)\prod_{j=1}^{p}p\left(y_{ij}|\theta ,z_{i},\rho_{j,z_{i}}\right),$$

where θ is the set of other parameters defined below.

### 2.2 | Prior and full conditional distributions

In our specific case with a two-dimensional latent structure, one for sample clustering $\left(Z\right)$ and the other for feature classification $\left(\rho \right)$, we are typically dealing with three separate entities for the vector of parameters θ for the model. That is, θ can be decomposed as, $\theta =\left(\text{Ω},\xi ,\zeta \right)$, where, (i) $\text{Ω}=\left\{\omega_{k};k=1,2,\ldots ,K\right\}$ and $\omega_{k}=\text{Prob}\left(z_{i}=k\right)$ is the cluster prior probability that an individual i belongs to cluster k, (ii) $\xi =\left\{\xi_{rs};r,s=1,2,3\right\}$ is the common transition matrix of the Markov chain process $\rho_{k}=\left(\rho_{1,k},\rho_{2,k},\ldots ,\rho_{p,k}\right)$ which is the sequence of states (1,2,3) of the p features, $\xi_{rs}$ is the transition probability from state r to state s, and (iii) $\zeta =\left(\mu_{jkl},\sigma_{jkl}^{2},\mu_{kl},\sigma_{kl}^{2},\sigma ;k=1,\ldots ,K;l=1,2;j=1,\ldots ,p\right)$ parameterizes the conditional distribution of $y_{ij}$ given z and ρ.

The initial distribution of the process $\rho_{k}=\left\{\rho_{j,k},j=1,\ldots ,p\right\}$ is assumed to be a uniform distribution, that is, $v=\left(v_{1},v_{2},v_{3}\right)$ with $v_{l}=P\left(\rho_{1,k}=l\right)=\frac{1}{3}$ for $l\in \left\{1,2,3\right\}$. We will impose conjugate Dirichlet prior both for estimating the cluster probabilities $\left(\text{Ω}\right)$ and transition probability matrix $\xi =\left(\xi_{rs}\right)$.

#### Cluster probabilities:

We impose a Dirichlet prior distribution for Ω with parameters $\left(\alpha_{1},\ldots ,\alpha_{K}\right)$ with $\alpha_{k}>0$. Hence, the full conditional of Ω is also a Dirichlet distribution with parameters $\left.\alpha_{1}+\sum_{i=1}^{n}I\left(z_{i}=1\right),\ldots ,\alpha_{K}+\sum_{i=1}^{n}I\left(z_{i}=K\right)\right)$ where $\sum_{k=1}^{K}\omega_{k}=1$ and $I\left(z_{i}=k\right)=1$ if $z_{i}=k$ and 0 otherwise.

#### Transition matrix:

We assume that each row $r\in \left\{1,2,3\right\}$ of ξ has a prior distribution that follows a Dirichlet in the following sense:$\left(\xi_{r1},\xi_{r2},\xi_{r3}\right)∼Dir_{3}\left(\delta_{1},\delta_{2},\delta_{3}\right)$. Then, given the Markov chain, $\rho_{k}=\left(\rho_{1,k},\rho_{2,k},\ldots \ldots ,\rho_{p,k}\right)$ for an arbitrary cluster k, each vector row of ξ follows also a Dirichlet distribution with parameters $\left(\delta_{1}+n_{r1},\delta_{2}+n_{r2},\delta_{3}+n_{r3}\right)$ where $n_{rl}$ denotes number of transitions from hidden state r→l and defined as

$$n_{rl}=\sum_{k=1}^{K}\sum_{j=1}^{p-1}I\left(\rho_{j,k}=r,\rho_{j+1,k}=l\right),\;\forall r,l\in 1,2,3.$$

#### Mean and variance parameters:

For every l=1,2, we assume $sign\left(\mu_{kl}\right)\mu_{kl}∼TN\left(t,\mu_{0},\sigma_{\mu_{0}}^{2}\right)$, a truncated normal prior with left truncation point t>0, mean $\mu_{0}$, and variance $\sigma_{\mu_{0}}^{2}$. $sign\left(\mu_{kl}\right)=1$ if $\mu_{kl}>0$ and −1 otherwise. The full conditional distribution of $\mu_{kl}$ is also a truncated normal

(4) $$sign\left(\mu_{kl}\right)\mu_{kl}|\theta ,\rho ,y,z∼TN\left(t,\frac{\frac{\mu_{0}\sigma_{kl}^{2}}{\sigma_{\mu_{0}}^{2}}+M_{kl}}{\frac{\sigma_{kl}^{2}}{\sigma_{\mu_{0}}^{2}}+N_{kl}},\frac{1}{\frac{1}{\sigma_{\mu_{0}}^{2}}+\frac{N_{kl}}{\sigma_{kl}^{2}}}\right),$$

where $M_{kl}=\sum_{j=1}^{p}I\left(\rho_{j,k}=l\right)\mu_{jkl}$ and $N_{kl}=\sum_{j=1}^{p}I\left(\rho_{j,k}=l\right)$, for $l\in \left\{1,2\right\}$ and $k\in \left\{1,2,\ldots ,K\right\}$.

Similarly, for the variance parameter $\sigma_{kl}^{2}$, we assume $\sigma_{kl}^{2}∼IG\left(\alpha_{0},\beta_{0}\right)$ which is an inverse-gamma distribution with fixed parameters $\left(\alpha_{0},\beta_{0}\right)$. The full conditional distribution of $\sigma_{kl}^{2}$ can be written as

(5) $$\sigma_{kl}^{2}|\theta ,\rho ,y,z∼IG\left(\alpha_{0}+\frac{N_{kl}}{2},\beta_{0}+\frac{S_{kl}^{2}}{2}\right),$$

where $S_{kl}^{2}=\sum_{j=1}^{p}{\left(\mu_{jkl}-\mu_{kl}\right)}^{2}I\left(\rho_{j,k}=l\right)$, for $l\in \left\{1,2\right\}$ and $k\in \left\{1,2,\ldots ,K\right\}$.

The full conditional of the error variance $\sigma_{j3}^{2}$ for irrelevant features is as

(6) $$\sigma_{j3}^{2}|\theta ,\rho ,y,z∼IG\left(\alpha_{0}+\frac{N_{\rho }^{\prime 2}}{2},\beta_{0}+\frac{S_{\rho }^{\prime 2}}{2}\right),$$

where $N_{\rho }^{\prime }=\sum_{i=1}^{n}\sum_{j=1}^{p}I\left(\rho_{j,z_{i}}=3\right)$ and $S_{\rho }^{\text{'}2}=\sum_{i=1}^{n}\sum_{j=1}^{p}I\left(\rho_{j,z_{i}}=3\right){\left(y_{ij}-\mu_{j,z_{i},3}\right)}^{2}$.

The full conditional of the error variance $\sigma_{jkl}^{2}$ for relevant features $\left(l\in \left\{1,2\right\}\right)$ is as

(7) $$\sigma_{jkl}^{2}|\theta ,\rho ,y,z∼IG\left(\alpha_{0}+\frac{N_{\rho }^{\text{''}}}{2},\beta_{0}+\frac{S_{\rho }^{\text{''}2}}{2}\right),$$

where $N_{kl}^{\text{''}}=\sum_{i=1}^{n}\sum_{j=1}^{p}I\left(\rho_{j,k}=l,z_{i}=k\right)$ and $S_{kl}^{\text{''}2}=\sum_{i=1}^{n}\sum_{j=1}^{p}I\left(\rho_{j,k}=l,z_{i}=k\right){\left(y_{ij}-\mu_{jkl}\right)}^{2}$.

The full conditional distribution of $\mu_{jkl}\left(l\in \left\{1,2\right\}\right)$ is a normal distribution

(8) $$\mu_{jkl}|\theta ,\rho ,y,z∼\text{Normal}\left(\frac{\frac{\mu_{kl}\sigma_{jkl}^{2}}{\sigma_{kl}^{2}}+M_{jkl}^{\text{'}}}{\frac{\sigma_{jkl}^{2}}{\sigma_{kl}^{2}}+N_{jkl}^{\text{'}}},\frac{1}{\frac{1}{\sigma_{kl}^{2}}+\frac{N_{jkl}^{\text{'}}}{\sigma_{jkl}^{2}}}\right),$$

$$\begin{array}{l} \underline{\text{ALGORITHM}\;\text{1}\;\text{Overview}\;\text{of}\;\text{MCMC}\;\text{Gibbs}\;\text{Sampling}\;\text{algorithm}\;\;\;\;\;\;\;\;\;\;\;\;\;\;\;\;\;\;\;\;\;\;\;\;\;\;\;\;\;\;\;\;\;\;\;\;\;\;\;\;\;\;\;\;\;\;\;\;\;\;\;\;\;\;\;\;\;\;\;\;\;\;\;\;\;\;\;\;\;\;\;\;\;\;\;\;\;\;\;\;\;\;\;\;\;\;\;\;\;\;\;\;\;\;\;\;\;\;\;\;\;\;\;\;\;\;\;\;\;\;\;\;\;\;\;\;\;\;\;\;\;\;\;\;\;\;\;\;\;\;\;\;\;\;\;\;\;\;\;\;\;\;\;\;\;\;\;\;\;\;\;\;}\\ \text{Input:}y_{1}\ldots y_{n},N:\text{Number of MCMC iterations}\\ \text{Output:MCMC samples of all parameters}\\ \text{1:}\;\text{Initialization:The latent clustering vector}\;z\;\text{and the latent chain}\;\rho_{k}\text{ are initialized using discrete uniform prior for}\;K\;\text{clusters and}\\ \;\text{ three groups, respectively, where the elements,}\\ \;\;\;\;\;\;\;\;\;z_{1},\ldots ,z_{n}{∼}^{\text{i.i.d}\;}multinomial_{K}\left(1,\frac{1}{K}\right)\;\text{and for every}\;j\;\text{and}\;k,\rho_{j,k}{∼}^{\text{i.i.d}\;}multinomial_{3}\left(1,\frac{1}{3}\right).\\ 2:\;\text{for}\;t\leftarrow 1\;\text{to}\;N\;\text{do}\\ 3:\;\text{Simulate the new probability vector}\;\text{Ω}\;\text{from its full conditional distribution}\;\left(\text{a Dirichlet distribution}\right)\text{, conditional on the previous}\;z\text{.}\\ \;\;\;\text{In the first iteration, the random initialized value of}\;z\;\text{is used as an input.}\\ 4:\;\text{New values for}\;\mu_{jkl}\;\text{and}\;\mu_{kl}\text{,}\;k=1,\ldots ,K,\;l=1,2,\;\text{and}\;j=1,\ldots ,p\;\text{are simulated from the full conditional distribution}\\ \;\;\;\left(\text{see Equations 8 and 4}\;\right)\text{.}\\ 6:\;\text{Simulate the rows of}\;\xi \;\text{independently, with the}\;r\text{ th row from the their full conditional Dirichlet distribution.}\\ 7:\;\text{New classification vector elements}\;z_{i}\text{,}\;i=1,2,\ldots .,n\;\text{is generated based on the following steps:}\\ \;\;\left(\text{i}\right)\text{Determine the posterior probability of observing}\;y_{i}\;\text{as belonging to a particular cluster}\;k\;\text{based on}\;\left(\text{11}\right)\\ \;\;\left(\text{ii}\right)\text{Simulate classification vector}\;\text{values}\;z_{i}\;\text{from the multinomial distribution determined in the above step}\left(\text{i}\right)\\ 8:\;\text{In the last step, simulate the latent chain}\;\rho_{j,k}\text{'s similarly as in Step 7 from their full conditional distribution (multinomial}\\ \;\;\;\text{distribution, see Equations 9 and 10).}\\ 9:\;\text{end for}\\ \underline{10:\text{return MCMC samples of all parameters.}\;\;\;\;\;\;\;\;\;\;\;\;\;\;\;\;\;\;\;\;\;\;\;\;\;\;\;\;\;\;\;\;\;\;\;\;\;\;\;\;\;\;\;\;\;\;\;\;\;\;\;\;\;\;\;\;\;\;\;\;\;\;\;\;\;\;\;\;\;\;\;\;\;\;\;\;\;\;\;\;\;\;\;\;\;\;\;\;\;\;\;\;\;\;\;\;\;\;\;\;\;\;\;\;\;\;\;\;\;\;\;\;\;\;\;\;\;\;\;\;\;\;\;\;\;\;\;\;\;\;\;\;\;\;\;\;\;\;\;\;\;\;\;\;\;\;\;\;\;\;\;\;\;\;\;\;\;\;\;\;\;\;\;\;\;\;\;\;\;\;\;\;\;\;\;\;\;\;\;\;\;\;\;\;\;\;\;\;\;\;\;\;\;\;\;} \end{array}$$

where

$$M_{jkl}^{\text{'}}=\sum_{i=1}^{n}I\left(\rho_{j,k}=l,z_{i}=k\right)y_{ij}$$

#### Latent chains $\rho_{k}$’s and latent cluster memberships z:

For each cluster k, the full conditional probabilities of ρ can be written as

(9) $$p\left(\rho_{1,k}=l|\zeta ,\rho_{2,k},y,z\right)\propto \xi_{l,\rho_{2k}}\prod_{i=1}^{n}I\left(z_{i}=k\right)\frac{1}{\sigma_{1kl}}\text{exp}\left\{-\frac{{\left(y_{i1}-\mu_{1kl}\right)}^{2}}{2\sigma_{1kl}^{2}}\right\},$$

(10) $$p\left(\rho_{j,k}=l|\zeta ,y,\rho_{\left(j-1,k\right)},\rho_{j+1,k},z\right)\propto \xi_{\rho_{j-1,k},l}\xi_{l,\rho_{j+1,k}}\prod_{i=1}^{n}I\left(z_{i}=k\right)\frac{1}{\sigma_{jkl}}\text{exp}\left\{-\frac{{\left(y_{ij}-\mu_{jkl}\right)}^{2}}{2\sigma_{jkl}^{2}}\right\}$$

for j=2,3,….,p and k=1,2,…,K. The full conditional probability for observation i belong to cluster k is

(11) $$p\left(z_{i}=k|\theta ,\rho ,y\right)\propto \omega_{k}\prod_{j=1}^{p}\prod_{l=1}^{3}I\left(\rho_{j,k}=l\right)\frac{1}{\sigma_{jkl}}\text{exp}\left\{-\frac{{\left(y_{ij}-\mu_{jkl}\right)}^{2}}{2\sigma_{jkl}^{2}}\right\}.$$

### 2.3 | Gibbs sampling scheme

The overview of our Gibbs sampling scheme is presented in Algorithm 1. After every iteration, the parameter values are updated and used in the next iteration.

The number of clusters K in the proposed work is fixed. To determine an optimal K, we used the deviance information criterion (DIC, Gelman et al., 2014) to measure both the goodness of fit of the model and the model complexity and it is defined as $DIC=\bar{D\left(\text{Θ}\right)}+p_{DIC}$, where $\text{Θ}=\left(\theta ,z,\rho \right)$ represents the set of unique parameters (including z and ρ), $\bar{D\left(\text{Θ}\right)}$ is the posterior mean of the deviance $D\left(\text{Θ}\right)$, and $p_{DIC}$ is the effective number of parameters used as a measure of model complexity. From the MCMC samples, the DIC is estimated as

$$DIC=-4\left[E_{\text{Θ|}y}\left\{\text{log}\;p\left(y|\text{Θ}\right)\right\}\right]+2\text{log}\;p\left(y|\tilde{\theta },\tilde{z},\tilde{\rho }\right)$$

$$\approx \frac{-4}{S}\sum_{s=1}^{S}\text{log}\;p\left(y|\theta^{s},z^{s},\rho^{s}\right)+2\text{log}\;p\left(y|\tilde{\theta },\tilde{z},\tilde{\rho }\right),$$

where S is the number of MCMC samples, $\left(\theta^{s},z^{s},\rho^{s}\right)$ is the s th MCMC sample of all parameters, $\left(\tilde{\theta },\tilde{z},\tilde{\rho }\right)$ is the joint maximum a posterior estimate (Geman & Geman, 1984) approximated by the MCMC output. Given a set of competing models, smaller DIC values indicate a better-fitting model. We will use this criterion for selecting the number of clusters K.

## 3 | SIMULATED DATA STUDY

### Setting 1:

**FIGURE 2:**
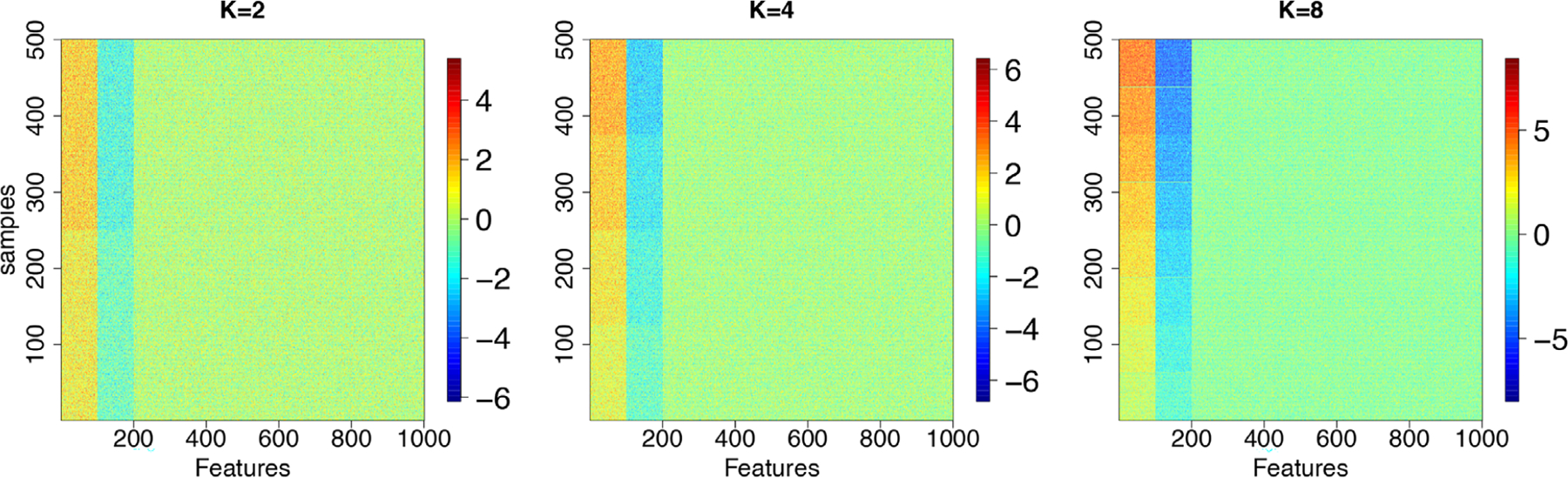
Images of some simulated data with respect to the number of clusters $K\in \left\{2,4,8\right\}$ when n=500 and $\sigma_{jkl}^{2}=1$.

We tested our method on 12 simulated test scenarios by dividing them into pairs for comparison. The difference between the paired scenarios is that the input variance to the data-generating process is doubled to 2.0 in scenarios 2, 4, 6, 8, 10, and 12 in comparison to their respective paired case. The model’s clustering capacity was tested by increasing (i) the number of clustering regimes, $K\in \left\{2,4,8\right\}$ and (ii) the number of subjects, $n\in \left\{100,500\right\}$ for the generated data input to each paired case. In this first setting, we assume that the first 100 features are *overexpressed* (i.e., $\rho_{j,k}=1$) in each sample cluster, the next 100 features are *underexpressed* (i.e., $\rho_{j,k}=2$). The remaining features $\left(p-200=800\right)$ are irrelevant for every sample cluster. As the features that belong to the same states are close (i.e., are ordered), we expect that our main approach that includes HMM would perform better. Expression values are generated as $y_{ij}=\mu_{kl}+\epsilon_{ijk}$, where $\mu_{k1}=\left(k+1\right)/2$, $\mu_{k2}=-\left(k+1\right)/2$, and $\mu_{k3}=0$ for every k=1,…,K. $\epsilon_{ijk}$ is normal with variance $\sigma_{jkl}^{2}\in \left\{1,2\right\}$. This will allow us to have different means between sample clusters. For cluster 1, for instance $\left(k=1\right)$, the means are $\mu_{11}=1$ and $\mu_{12}=-1$ for overexpressed and underexpressed features, respectively. Figure 2 shows some examples of simulated data for $K\in \left\{2,4,8\right\}$.

### Setting 2:

In this setting, the relevant 200 features are now chosen randomly out of the p=1000 features. Relevant features within sample clusters are not necessarily ordered as in Setting 1. Hence, we do not expect an impact on clustering of the order structure between features for this scenario, that is, we would not expect an improvement of the feature clustering performance within sample clusters with the use of the Hidden Markov structure that encourages "closer" features to be clustered together in one of the three feature groups (underexpressed, overexpressed, and irrelevant). The rest of the parameters for simulated data in this setting are set as in Setting 1.

### Hyperparameter settings:

In our Bayesian approach, we specify the prior distributions for each model parameter, in this case, cluster probabilities $\left(\text{Ω}\right)$, feature transition probability vector $\left(\xi \right)$, mean parameter for each cluster ($\mu_{kl}$ and $\mu_{jkl}$), and also the cluster variance parameter ($\sigma_{kl}^{2}$ and $\sigma_{jkl}^{2}$). We set hyperparameters of those prior distributions as follows. A truncated Normal distribution was used as a prior for cluster mean parameter $\left(\mu_{kl}\right)$, which was lower truncated at t=0.2 for selected overexpressed conditions, and, upper truncated at −t for underexpressed conditions. The hyperparameters were set to $\mu_{0}=0.0$ and $\sigma_{\mu_{0}}^{2}=1000$ for the mean and variance of the distribution, respectively. The mean was kept constant at zero when estimating biclusters corresponding to irrelevant features, $\left(\rho_{j,k}=3\right)$. For estimating the cluster variance parameters ($\sigma_{kl}^{2}$ and $\sigma_{jkl}^{2}$), the hyperparameters were set to $\alpha_{0}=1$ and $\beta_{0}=1$ for scale and shape of the distribution, respectively. We also fit the model when $\alpha_{0}=0.1$ and $\beta_{0}=0.1$, a "more" noninformative prior for the variance parameters. Results are compared in Section 3.2. We choose natural noninformative Dirichlet prior for estimating cluster and transition probabilities, which is to set $\alpha_{k}=1$ for k=1,2,…,K and $\delta_{l}=1$ for l=1,2,3.

To study the importance of incorporating the order structure and the biological significance (over/underexpressed features) into the determination of biclusters, we fitted four different models derived from our algorithm: (i) HMMBi-C: this method determines biclusters by assuming an order structure between features through a hidden Markov structure on the data y, and imposing a constraint on the mean parameters $\mu_{kl}$ (positive, negative, and zero depending on the feature state) in order to have a better biological interpretation of obtained clusters; (ii) HMMBi-NoC: this method assumes an order structure between features through a hidden Markov structure on the data y, but does not impose a constraint on the mean parameters $\mu_{kl}$; (iii) NoHMMBi-C: this method does not assume an order structure between features, but does impose a constraint on the mean parameters $\mu_{kl}$; and (iv) NoHMMBi-NoC: this method does not assume an order structure between features, and does not impose a constraint on the mean parameters $\mu_{kl}$. Feature labels $\rho_{j,k}\in \left\{1,2\right\}$ are not identifiable anymore for nonconstraint methods (i.e., HMMBi-NoC and NoHMMBi-NoC) within every cluster k but we would expect that the two feature clusters 1 and 2 obtained from these methods are exclusively each either the underexpressed feature cluster or the overexpressed feature cluster. We will also compare these four model methods with four other relevant (bi)clustering methods. We will use the R-package biclust (Kaiser et al., 2018) to compare with two among the several other provided algorithms to find biclusters in two-dimensional data, namely, *BC-Spectral* which is based on Spectral Bicluster algorithm (Kaiser et al., 2018) as described in Kluger et al. (2003) and *BC-Plaid* which performs Plaid Model Biclustering as described in Turner et al. (2005). We will also compare with model-based biclustering technique *FABIA* (Hochreiter et al., 2010) which uses factor analysis for determining biclusters. Finally, we compared our methods with the penalized biclustering method (*PenPlaid*) described in Chekouo and Murua (2015).

#### 3.1 | Model comparison

The principal approaches used in clustering comparison can be described through their development of criteria, of which there are two main approaches: pair counting (Pfitzner et al., 2008) and information-theoretic. In order to compare clustering results, we will use one of the variations of the pair counting measure the so-called $F_{1}$ measure which has been extensively used in the text mining literature and introduced to the biclustering literature. Consider two biclusters A and B, the $F_{1}$ measure (Chekouo & Murua, 2015) between A and B is defined as:

$$F_{1}\left(A,B\right)=\frac{2\left(r_{A\cap B}\right)\times \left(c_{A\cap B}\right)}{n_{A}+n_{B}}=2/\left(1/recall+1/precision\right),$$

where $recall=\frac{\left(r_{A\cap B}\right)\left(c_{A\cap B}\right)}{n_{B}}$ and $precision=\frac{\left(r_{A\cap B}\right)\left(c_{A\cap B}\right)}{n_{A}}$,

$r_{A\cap B}$ denotes the number of common genes, $c_{A\cap B}$ number of common conditions, and, $n_{A}\left(=r_{A}c_{A}\right)$, $n_{B}\left(=r_{B}c_{B}\right)$ the number of elements in biclusters A and B, respectively. The $F_{1}$ measure is based on the harmonic mean of precision and recall, where recall measures the proportion of elements in B that belong to A and precision measures the proportion of elements in A captured in B. When several biclusters are to be compared we will use an $F_{1}$-type average. Let $M_{1}=\left\{A_{1},A_{2},\ldots \;\ldots \;.,A_{K}\right\}$ be the set of estimated biclusters, and $M_{2}=\left\{B_{1},B_{2},\ldots \;\ldots \;.,B_{L}\right\}$, the set of true biclusters. Then, the measure of the similarity of the estimate $M_{1}$ to the true biclustering $M_{2}$ is given by

$$S\left(M_{1},M_{2}\right)=\frac{1}{K}\sum_{k=1}^{K}\max_{j}F_{1}\left(A_{k},B_{j}\right).$$

Our $F_{1}$-type average is a symmetrized version of $S\left(M_{1},M_{2}\right)$ and will be defined as $F_{1}\left(M_{1},M_{2}\right)=\left(S\left(M_{1},M_{2}\right)+\right.\left.S\left(M_{2},M_{1}\right)\right)/2$ (Murua & Quintana, 2022). Note that $F_{1}\left(M_{1},M_{2}\right)\leq 1$, and it is equal to 1.0 if $M_{1}=M_{2}$.

The above-defined $F_{1}$ measure will be employed to compare our model methods with other relevant biclustering methods.

#### 3.2 | Results

Table 1 displays the means and standard deviations of the $F_{1}$ measure comparing the true collections of (bi)clusters to what is estimated by each of the methods studied for Setting 1 where features are generated from an ordering structure. Our main proposed model HMMBi-C, and the other three models are obtained by adjusting our main model. For the small data sets (n=100, scenarios 1–6), the HMMBi-C model’s performance is very similar to that of HMMBi-NoC which does not assume a constraint on the cluster parameters. However, when n is large (scenarios 7–12), HMMBi-C performs slightly better than HMMBi-NoC; that is, incorporating the constraint in the HMM model provides better performance. Moreover, for every scenario, HMMBi-C performs much better than models (NoHMMBi-C and NoHMMBi-C) that do not incorporate an ordering structure of features. Adding a constraint on a model without the HMM structure (NoHMMBi-C) has also better performance than not adding them (NoHMMBi-NoC), in particular in scenarios 3, 7, 8, and 10. In addition, as expected, when K or $\sigma_{jkl}^{2}$ increases, each method’s performance goes down. Overall, HMMBi-C clearly performs better than the three other methods, which shows that we obtain better results when we incorporate the ordering structure (through HMM) and a constraint on the cluster parameters for biological interpretation. Table 2 shows the results when $\alpha_{0}=\beta_{0}=0.1$. They are slightly worse than the results when $\alpha_{0}=\beta_{0}=1$ (Table 1) for almost all scenarios. In addition, the method HMMBi-C also performs much better in almost all scenarios when $\alpha_{0}=\beta_{0}=0.1$. Table 3 displays results obtained from fitting some competing biclustering algorithms on the simulated data. For a fair comparison with our previous four methods, we fix the number of biclusters to 3×K. First, all those methods perform poorly compared to our four methods mentioned previously. Second, among the competing methods, the penalized method of Chekouo and Murua (2015) performs much better than the others. The plaid model of Turner et al. (2005) (BC-Plaid) were not able to identify any biclusters in most of the scenario. Also, *BC-Spectral* has not identified any biclusters for every scenario (results not shown in the table).

Table 4 reports the results of Setting 2 of the simulated data, where the features are not ordered and assigned randomly to the three different groups (underexpressed, overexpressed, and irrelevant) as described previously. As expected, Table 4 shows that methods with ordering structure assumption perform similarly to methods without ordering structure (HMMBi-C vs. NoHMMBi-C or HMMBi-NoC vs. NoHMMBi-C). However, there is a slight improvement for models with constraints when compared to models without constraints (HMMBi-C vs. HMMBi-NoC or NoHMMBi-C vs. NoHMMBi-NoC).

**FIGURE 3:**
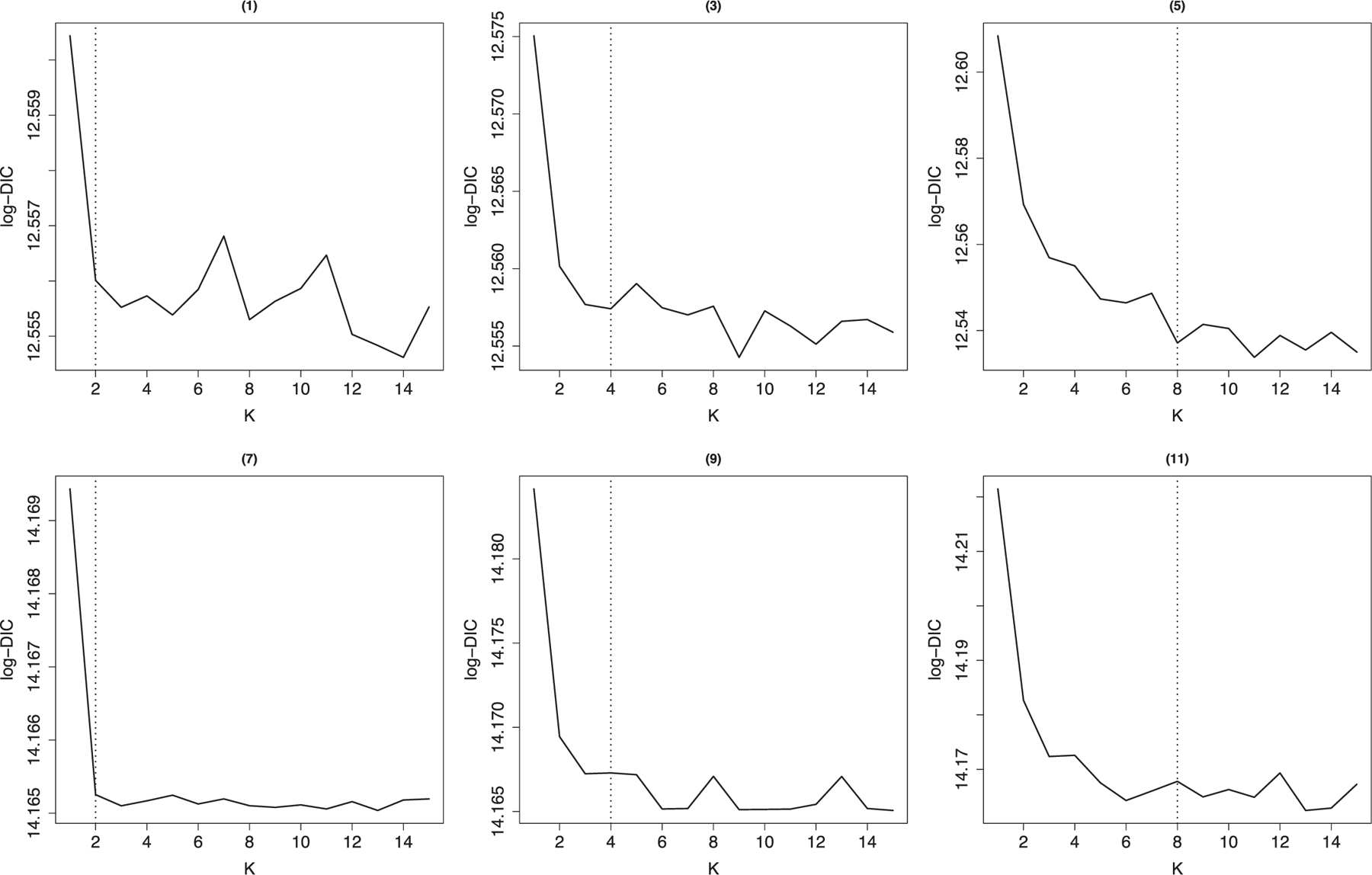
(Log-)DIC (deviance information criterion) measures for scenarios 1, 3, 5, 7, 9, and 11 where $\sigma_{jkl}^{2}=1$ for model HMMBi-C. The horizontal dot lines correspond to the true number of clusters.

Figure 3 plots DIC measures estimate for K=1 to 15 for scenarios where the error variance is 1. As we notice from the plots, when more and more clusters are added, the DIC values begin to decline gradually until around the true number of clusters where the DIC values become relatively constant. We can then use a rule of thumb to select K associated with a point in the flat part of the DIC curve that falls near the elbow of the curve. We also observed that after the true number of clusters, some clustering results have clusters with few or zero subjects, and the corresponding number of clusters of these clustering results should not be selected.

## 4 | APPLICATION TO TCGA KIDNEY mRNA EXPRESSION DATA

We applied our new methodology to 534 samples from TCGA KIRC (kidney renal cell carcinoma) data, using the mRNA expression information collected from the Illumina HiSeq2000 platform (~20,000 protein-coding genes). We removed genes (i) with more than 30% missing values, and we imputed the remaining missing values with the k-nearest neighbor method implemented in the R package *impute*, and (ii) with minimal variance. We used GO annotation to compute semantic similarity score between each gene pair by using the *getGeneSim* method provided with the Bioconductor package *GOSim*. We then reordered data frame columns (genes) based on a sorted genes list computed using the GO similarity score matrix computed from the Biological Process (BP) ontology. We retained 1009 mRNAs expressed across 534 samples and applied our methodology to the log-transformed standardized data. To run our MCMC algorithm, we used the same hyperparameter settings as in the simulation studies with 10,000 iterations and 5000 values discarded as "burn-in" for each run.

### 4.1 | Results

**FIGURE 4:**
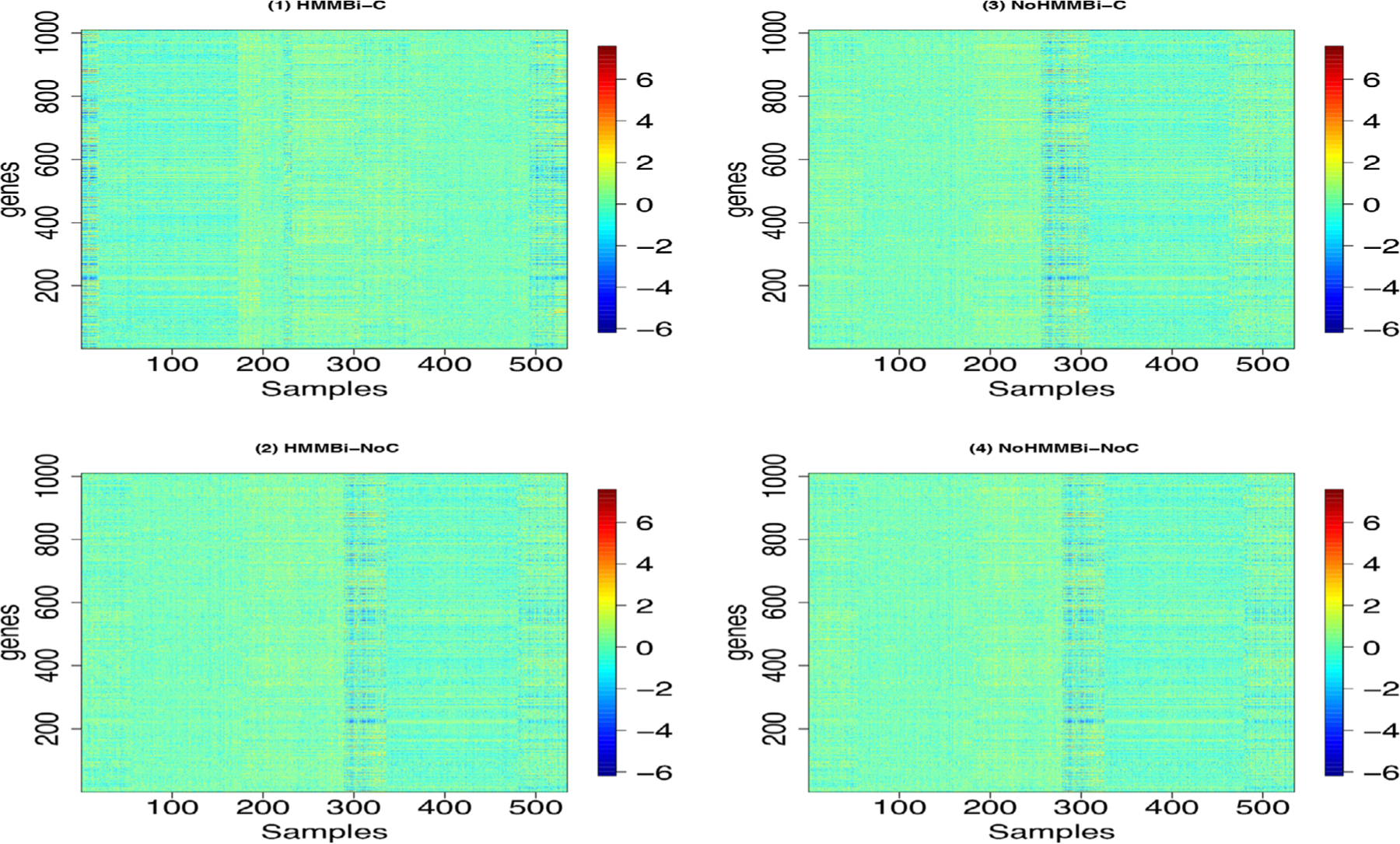
Images of observed data sorted with respect to each cluster for each of our four methods.

**FIGURE 5:**
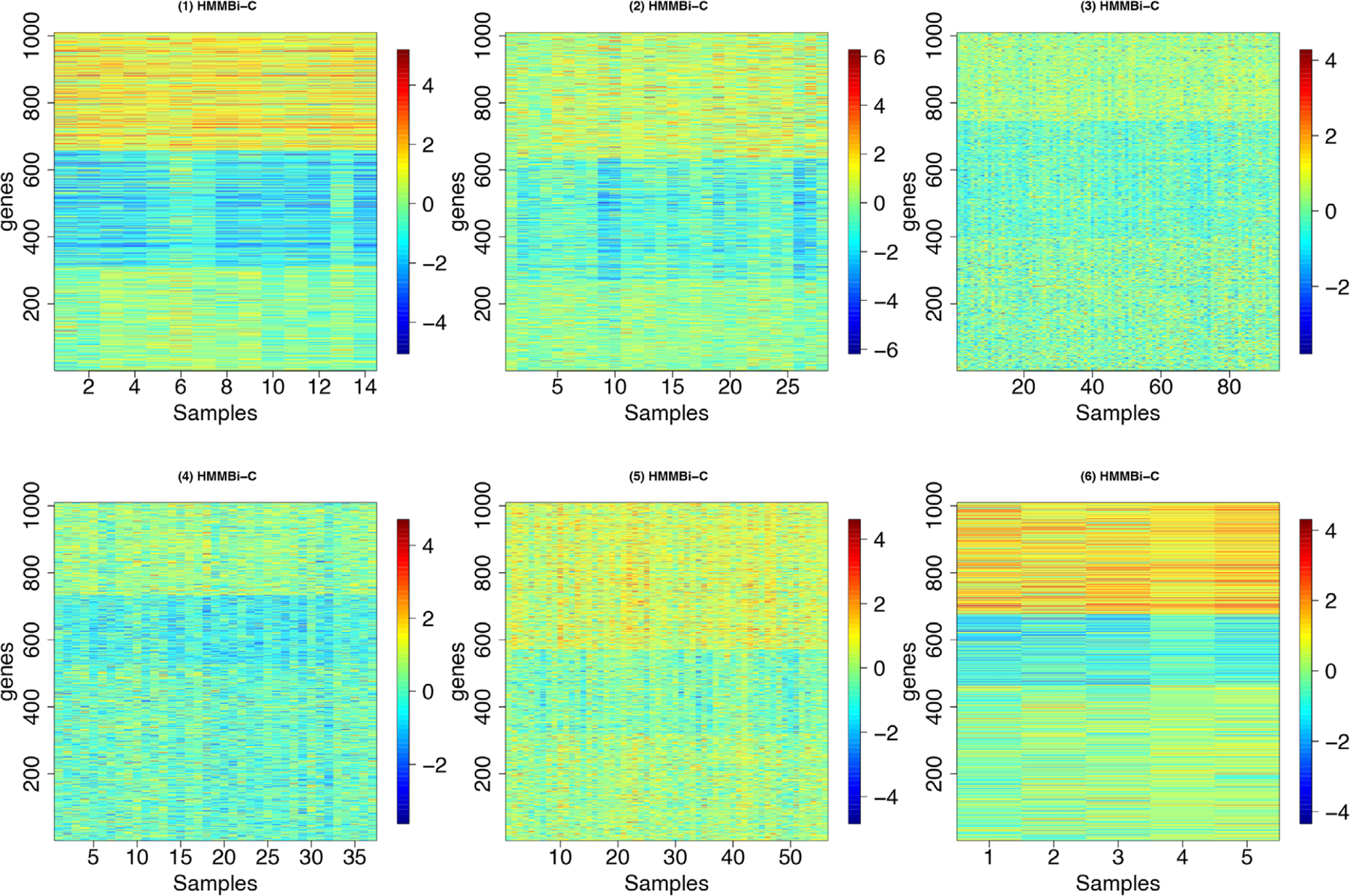
Images of clusters with features (genes) sorted with respect to overexpressed, underexpressed, and irrelevant groups of features. The six images represent images of the six first clusters identified using HMMBi-C.

We applied our four methods to the RNA-seq data described previously with the same hyperparameter settings as in the simulation study. Figure 4 shows images of our data sorted with respect to cluster samples of the four methods. It shows horizontal patterns to the images which correspond to clusters. DIC criteria for each method detected 13, 12, 10, and 9 clusters for the four methods HMMBi-C, HMMBi-NoC, NoHMMBi-C, and NoHMMBi-NoC, respectively. We discard clustering results with cluster sizes less than 3. Figure 5 shows the image of six clusters (out of 13) obtained using the HMMBi-C method. For each cluster image, we sorted the genes with respect to overexpressed genes, underexpressed, and irrelevant genes as classified by our method. The images show a clear distinction between the three groups of features. Table 5 shows the distribution of the three groups of features for each cluster. Overall, the number of underexpressed genes is usually less than the number of overexpressed and irrelevant genes. Multiple genes can be over (under)expressed in multiple clusters. For instance, 157 and 216 genes are over- and underexpressed in both clusters 1 and 2. In addition, the table shows cluster sample means estimated over under- and overexpressed genes, respectively, for each cluster. In particular, cluster 1 has the smallest mean for underexpressed genes $\left(\mu_{12}=-1.13\right)$, and cluster 6 has the largest "overexpressed" mean $\left(\mu_{61}=1.09\right)$.

To determine whether our clustering schemas are associated with survival outcomes (70% of observations are censored), for each cluster, we fitted multivariate Cox regressions with covariates as clusters obtained from the HMMBi-C clustering algorithm. We adjusted the model with clinical covariates such as age, sex, and cancer stages. Clusters 2 and 11 provide positive and negative associations with survival time with respective hazard ratios of 2.98 and 0.48. Both regressions have good predictive performance, with concordance indices of about 0.72.

**FIGURE 6:**
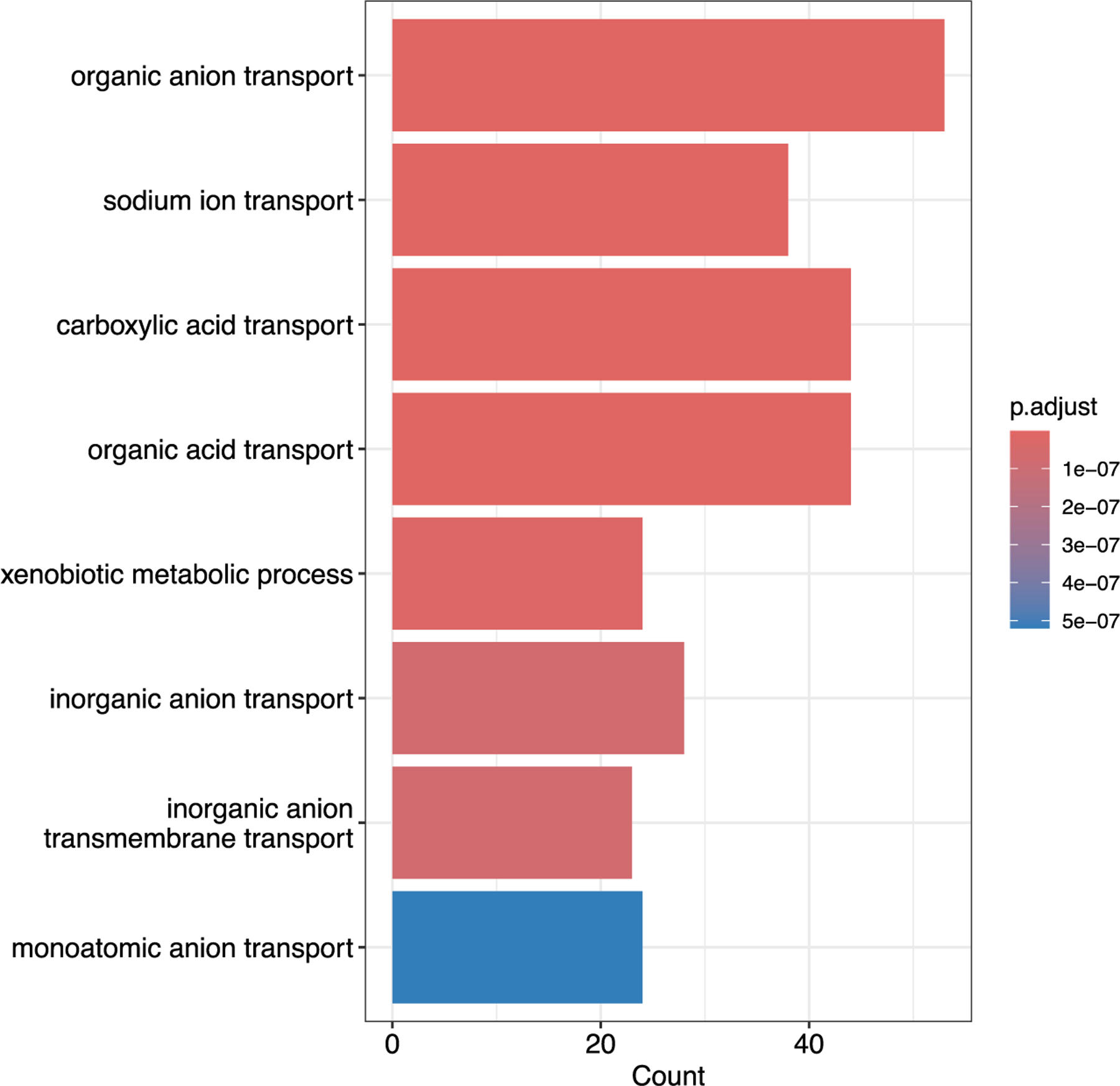
Top eight enriched Gene Ontology (GO) terms of GO Enrichment analysis of the gene set of over- and underexpressed genes in cluster 1 obtained from HMMBi-C.

**FIGURE 7:**
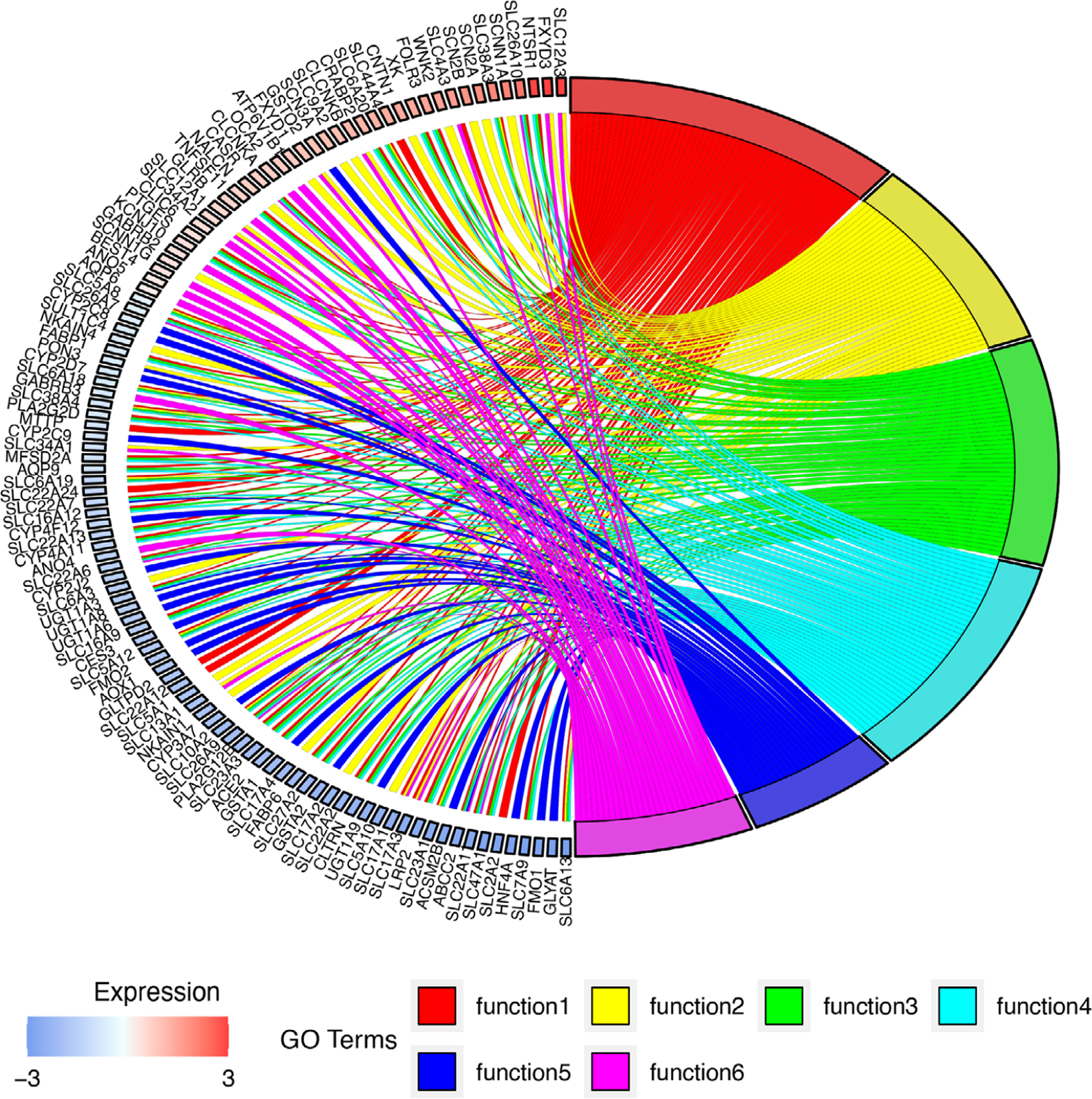
Gene Ontology (GO) Enrichment analysis of the gene set of over- and underexpressed genes in cluster 1 obtained from HMMBi-C. Top four GO terms: function1: anion transmembrane transport, function2: organic anion transport, function3: sodium ion transport, and function4: xenobiotic metabolic process.

GO enrichment analysis was performed on the over- and underexpressed genes from Cluster 1 of the method HMMBi-C. The top eight enriched GO terms are shown in Figure 6 with their respective counts in Cluster 1. Among them, GO terms *xenobiotic metabolic process* and *organic anion transport* (OAT) were dominant among underexpressed genes (see Figure 7). But the GO term *organic acid transport* seems to be equally distributed between the two groups (over- and underexpressed) of genes. As an example, OATs are active transport proteins that regulate anion balance in the body and are members of the superfamily SLC (solute carriers). They are primarily expressed in the kidney and liver and have been recently involved in renal cell carcinoma (Whisenant & Nigam, 2022).

## 5 | CONCLUSION

In this work, we proposed an innovative interpretable mixture modeling in the context of biclustering expression data. The method brings together feature selection and clustering simultaneously under a single "wrapper" method (Dy & Brodley, 2004; Law et al., 2004). This approach has a benefit from two standpoints; first, both the processes, feature selection, and sample clustering are integrated into the model fitting process in a unified procedure. Second, feature selection is performed within each cluster of samples and features can be classified automatically into three biological groups: underexpressed, overexpressed, and irrelevant. Features are classified using an HMM with initial ordering among the features imposed using prior biological knowledge (e.g., GO). Hence, for each cluster of samples, "close" features are encouraged to be in the same group.

We assumed here a mixture of normal distributions to model clusters with a fixed K, the number of clusters. For future work, we can explore a natural extension to other distributions/assumptions (e.g., t-distributions, infinite mixture of factor analysis, nonparametric, etc.) that would capture more complex structures with an unknown number of clusters.

Another essential step will be integrating other omics data types such as DNA-methylation or microRNA data with the available RNA-Seq data. Fortunately, TCGA consortium (our data source) provides samples matched between those omics data for various cancers. Hence, extending our methods to integrate multiomics data types is possible, but we would need to understand the association between those data. For instance, the association between methylation and gene expression is usually unknown and difficult to determine (Ma et al., 2017).

## Supplementary Material

Supporting information

## Figures and Tables

**TABLE 1:** Setting 1: Simulation results for all scenarios (1–12) across 20 replicates. Values reported are the averaged $F_{1}$ measures across 20 replicates. Standard errors are between parentheses. The highest values are in bold.

Scenario	n	K	$\sigma_{jkl}^{2}$	HMMBi-C	HMMBi-NoC	NoHMMBi-C	NoHMMBi-NoC
1	100	2	1	0.998 (0.001)	**0.999** (0.001)	0.982 (0.017)	0.981 (0.017)
2	100	2	2	0.527 (0.002)	**0.529** (0.001)	0.507 (0.001)	0.506 (0.000)
3	100	4	1	0.831 (0.014)	**0.833** (0.013)	0.769 (0.021)	0.721 (0.019)
4	100	4	2	**0.533** (0.004)	0.531 (0.003)	0.283 (0.003)	0.283 (0.003)
5	100	8	1	0.620 (0.012)	**0.647** (0.018)	0.519 (0.016)	0.54 (0.016)
6	100	8	2	0.393 (0.011)	**0.402** (0.012)	0.207 (0.006)	0.196 (0.007)
7	500	2	1	**0.983** (0.017)	0.930 (0.031)	0.940 (0.028)	0.926 (0.03)
8	500	2	2	**0.505** (0.001)	0.479 (0.012)	0.488 (0.006)	0.442 (0.017)
9	500	4	1	**0.855** (0.009)	0.832 (0.017)	0.654 (0.012)	0.590 (0.014)
10	500	4	2	**0.591** (0.019)	0.584 (0.024)	0.502 (0.011)	0.480 (0.011)
11	500	8	1	**0.713** (0.017)	0.710 (0.013)	0.716 (0.015)	0.708 (0.016)
12	500	8	2	**0.473** (0.007)	0.460 (0.010)	0.418 (0.013)	0.406 (0.016)

**TABLE 2:** Setting 1: Simulation results for all scenarios (1–12) across 20 replicates when $\alpha_{0}=\beta_{0}=0.1$. Values reported are the averaged $F_{1}$ measures across 20 replicates. Standard errors are between parentheses. The highest values are in bold.

Scenario	n	K	$\sigma_{jkl}^{2}$	HMMBi-C	HMMBi-NoC	NoHMMBi-C	NoHMMBi-NoC
1	100	2	1	0.856 (0.045)	0.795 (0.050)	**0.893** (0.037)	0.661 (0.045)
2	100	2	2	0.518 (0.001)	0.505 (0.008)	**0.642** (0.013)	0.563 (0.024)
3	100	4	1	**0.776** (0.023)	0.747 (0.027)	0.713 (0.023)	0.691 (0.027)
4	100	4	2	**0.484** (0.030)	0.427 (0.031)	0.336 (0.013)	0.283 (0.013)
5	100	8	1	**0.567** (0.014)	0.499 (0.015)	0.480 (0.021)	0.440 (0.017)
6	100	8	2	**0.341** (0.009)	0.332 (0.010)	0.234 (0.014)	0.239 (0.013)
7	500	2	1	**0.600** (0.044)	0.550 (0.038)	0.562 (0.029)	0.563 (0.028)
8	500	2	2	**0.518** (0.018)	0.436 (0.018)	0.514 (0.015)	0.485 (0.007)
9	500	4	1	**0.582** (0.023)	0.544 (0.028)	0.514 (0.004)	0.494 (0.011)
10	500	4	2	0.560 (0.016)	**0.561** (0.014)	0.542 (0.012)	0.494 (0.010)
11	500	8	1	**0.616** (0.010)	0.586 (0.012)	0.557 (0.007)	0.550 (0.010)
12	500	8	2	**0.436** (0.016)	0.470 (0.020)	0.383 (0.011)	0.344 (0.010)

**TABLE 3:** Setting 1: Simulation results for all scenarios (1–12) across 20 replicates. Values reported are the averaged $F_{1}$ measures across 20 replicates. Standard errors are between parentheses. Competing methods: BC-Plaid, FABIA, and PenPlaid.

Scenario	n	K	$\sigma_{jkl}^{2}$	BC-Plaid	FABIA	PenPlaid
1	100	2	1	–	0.177 (0.001)	0.363 (0.013)
2	100	2	2	–	0.163 (0.003)	0.371 (0.004)
3	100	4	1	–	0.087 (0.002)	0.277 (0.007)
4	100	4	2	–	0.030 (0.001)	0.253 (0.004)
5	100	8	1	0.084 (0.003)	0.044 (0.001)	0.238 (0.006)
6	100	8	2	–	0.042 (0.001)	0.278 (0.010)
7	500	2	1	–	0.167 (0.001)	0.389 (0.009)
8	500	2	2	–	–	0.359 (0.014)
9	500	4	1	–	–	0.334 (0.008)
10	500	4	2	–	–	0.277 (0.004)
11	500	8	1	0.095 (0.004)	0.042 (0.001)	0.235 (0.003)
12	500	8	2	0.000 (0.000)	–	0.209 (0.010)

**TABLE 4:** Setting 2: Simulation results for all scenarios (1–12) across 20 replicates. Values reported are the averaged F1 measures across 20 replicates. Standard errors are between parentheses. The highest values are in bold.

Scenario	n	K	$\sigma_{jkl}^{2}$	HMMBi-C	HMMBi-NoC	NoHMMBi-C	NoHMMBi-NoC
1	100	2	1	**0.982 (0.017)**	**0.982 (0.017)**	0.966 (0.023)	0.981 (0.017)
2	100	2	2	**0.507 (0.001)**	0.484 (0.01)	0.506 (0.001)	0.505 (0.001)
3	100	4	1	0.700 (0.016)	0.701 (0.022)	**0.712 (0.018)**	0.710 (0.018)
4	100	4	2	**0.284 (0.002)**	0.269 (0.007)	0.281 (0.002)	0.281 (0.002)
5	100	8	1	**0.510 (0.015)**	0.485 (0.013)	0.490 (0.015)	0.488 (0.02)
6	100	8	2	0.196 (0.006)	0.184 (0.010)	**0.198 (0.002)**	0.189 (0.004)
7	500	2	1	0.995 (0.003)	0.874 (0.025)	**0.997 (0.002)**	0.960 (0.013)
8	500	2	2	**0.633 (0.059)**	0.488 (0.042)	0.613 (0.049)	0.517 (0.048)
9	500	4	1	**0.859 (0.020)**	0.759 (0.031)	0.757 (0.020)	0.769 (0.022)
10	500	4	2	**0.499 (0.008)**	0.451 (0.013)	0.490 (0.007)	0.457 (0.012)
11	500	8	1	**0.771 (0.011)**	0.743 (0.017)	0.748 (0.016)	0.720 (0.019)
12	500	8	2	**0.417 (0.013)**	0.397 (0.019)	0.417 (0.01)	0.407 (0.011)

**TABLE 5:** The estimated cluster means and the distribution of the three groups of gene within each cluster obtained from HMMBi-C. The last column are p-values (nonadjusted) of the effect of each cluster on the survival time of kidney cancer.

Cluster	$\mu_{k1}$	$\mu_{k2}$	Over	Under	Irrelevant	*P*-Value (surv)
1	0.96	−1.13	352	348	309	0.7882
2	0.51	−0.88	376	362	271	<0.0000*
3	0.21	−0.21	264	346	399	0.6834
4	0.41	−0.31	275	206	528	0.124
5	0.48	−0.37	438	252	319	0.3522
6	1.09	−0.63	332	212	465	0.9831
7	0.65	−0.24	488	253	268	0.1583
8	0.43	−0.53	300	343	366	0.245
9	0.45	−0.22	278	126	605	0.7867
10	0.55	−0.21	500	33	476	0.148
11	0.22	−0.46	264	601	144	0.0048*
12	0.38	−0.55	235	489	285	0.4559
13	0.49	−1.09	280	471	258	0.8103

Asterisk means that the *p*-Value is less than 0.05.

## Data Availability

The data that support the findings of this study in Sections 3 and 4 are available in the R package *BiclustBHMM* at https://github.com/chekouo/BiclustBHMM. The observed data, as well as the simulation data process, are also available in the R package.

## References

[R1] Alon U, Barkai N, Notterman DA, Gish K, Ybarra S, Mack D, & Levine AJ (1999). Broad patterns of gene expression revealed by clustering analysis of tumor and normal colon tissues probed by oligonucleotide arrays. Proceedings of the National Academy of Sciences of the USA, 96(12), 6745–6750. 10359783 10.1073/pnas.96.12.6745 PMC21986

[R2] Chekouo T, & Murua A (2015). The penalized biclustering model and related algorithms. Journal of Applied Statistics, 42(6), 1255–1277.

[R3] Chekouo T, & Murua A (2018). High-dimensional variable selection with the plaid mixture model for clustering. Computational Statistics, 33(3), 1475–1496.

[R4] Chekouo T, Murua A, & Raffelsberger W (2015). The Gibbs-plaid biclustering model. Annals of Applied Statistics, 9(3), 1643–1670.

[R5] Cheng Y, & Church GM (2000). Biclustering of expression data. Proceedings International Conference on Intelligent Systems for Molecular Biology, 8, 93–103. 10977070

[R6] Cho H, Dhillon I, Guan Y, & Sra S (2004). Minimum sum-squared residue co-clustering of gene expression data. In International Conference on Data Mining (pp. 699–705).

[R7] Christophe D, & Nives Š (2017). The gene ontology handbook. Springer.

[R8] Cui S, Guha S, Ferreira MAR, & Tegge AN (2015). hmmSeq: A hidden Markov model for detecting differentially expressed genes from RNA-seq data. Annals of Applied Statistics, 9(2), 901–925.

[R9] Dy JG, & Brodley CE (2004). Feature selection for unsupervised learning. Journal of Machine Learning Research, 5, 845–889.

[R10] Fop M, & Murphy TB (2018). Variable selection methods for model-based clustering. Statistics Surveys, 12, 18–65.

[R11] Gelman A, Hwang J, & Vehtari A (2014). Understanding predictive information criteria for Bayesian models. Statistics and Computing, 24(6), 997–1016.

[R12] Geman S, & Geman D (1984). Stochastic relaxation, Gibbs distributions, and the Bayesian restoration of images. IEEE Transactions on Pattern Analysis and Machine Intelligence, 6(6), 721–741. 22499653 10.1109/tpami.1984.4767596

[R13] Getz G, Levine E, & Domany E (2000). Coupled two-way clustering analysis of gene microarray data. Proceedings of the National Academy of Sciences of the USA, 97, 12079–12084. 11035779 10.1073/pnas.210134797 PMC17297

[R14] Gu J, & Liu S (2008). Bayesian biclustering of gene expression data. BMC Genomics, 9(1), 113–120. 18366617 10.1186/1471-2164-9-S1-S4 PMC2386069

[R15] Hochreiter S, Bodenhofer U, Heusel M, Mayr A, Mitterecker A, Kasim A, Khamiakova T, Van Sanden S, Lin D, Talloen W, Bijnens L, G"ohlmann HWH, Shkedy Z, & Clevert D-A (2010). FABIA: Factor analysis for bicluster acquisition. Bioinformatics, 26(12), 1520–1527. 10.1093/bioinformatics/btq227 20418340 PMC2881408

[R16] Kaiser S, Santamaria R, Khamiakova T, Sill M, Theron R, Quintales L, Leisch F, & De Troyer E (2018). biclust: BiCluster Algorithms. R Package Version 2.0.1.

[R17] Kluger Y, Basri R, Chang JT, & Gerstein M (2003). Spectral biclustering of microarray data: Coclustering genes and conditions. Genome Research, 13(4), 703–716. 12671006 10.1101/gr.648603 PMC430175

[R18] Law MHC, Figueiredo MAT, & Jain AK (2004). Simultaneous feature selection and clustering using mixture models. IEEE Transactions on Pattern Analysis and Machine Intelligence, 26(9), 1154–1166. 15742891 10.1109/TPAMI.2004.71

[R19] Lazzeroni L, & Owen A (2002). Plaid models for gene expression data. Statistica Sinica, 12(1), 61–86.

[R20] Lee M, Shen H, Huang JZ, & Marron J (2010). Biclustering via sparse singular value decomposition. Biometrics, 66(4), 1087–1095. 20163403 10.1111/j.1541-0420.2010.01392.x

[R21] Li F, Yu G, Wang S, Bo X, Wu Y, & Qin Y (2010). GOSemSim: An R package for measuring semantic similarity among GO terms and gene products. Bioinformatics, 26(7), 976–978. 20179076 10.1093/bioinformatics/btq064

[R22] Ma X, Liu Z, Zhang Z, Huang X, & Tang W (2017). Multiple network algorithm for epigenetic modules via the integration of genome-wide DNA methylation and gene expression data. BMC Bioinformatics, 18(1), 72. 28137264 10.1186/s12859-017-1490-6 PMC5282853

[R23] Maugis C, Celeux G, & Martin-Magniette ML (2009). Variable selection in model-based clustering: A general variable role modeling. Computational Statistics & Data Analysis, 53(11), 3872–3882.

[R24] Murua A, & Quintana FA (2022). Biclustering via semiparametric Bayesian inference. Bayesian Analysis, 17(3), 969–995.

[R25] Pfitzner D, Leibbrandt R, & Powers D (2008). Characterization and evaluation of similarity measures for pairs of clusterings. Knowledge and Information Systems, 19(3), 361–394.

[R26] Sill M, Kaiser S, Benner A, & Kopp-Schneider A (2011). Robust biclustering by sparse singular value decomposition incorporating stability selection. Bioinformatics (Oxford, England), 27, 2089–2097. 21636597 10.1093/bioinformatics/btr322

[R27] Tanay A, Sharan R, & Shamir R (2002). Discovering statistically significant biclusters in gene expression data. Bioinformatics, 18(Suppl_1), S136–S144. 12169541 10.1093/bioinformatics/18.suppl_1.s136

[R28] The Cancer Genome Atlas Research Network, Creighton CJ, Morgan M, Gunaratne PH, Wheeler DA, Gibbs RA, Gordon Robertson A, Chu A, Beroukhim R, Cibulskis K, Signoretti S, Vandin Hsin-Ta Wu F, Raphael BJ, Verhaak RGW, Tamboli P, Torres-Garcia W, Akbani R, Weinstein JN, Reuter V, … Sofia HJ (2013). Comprehensive molecular characterization of clear cell renal cell carcinoma. Nature, 499, 43–49. 23792563 10.1038/nature12222 PMC3771322

[R29] Thomas PD, Wood V, Mungall CJ, Lewis SE, & Blake JA (2012). On the use of gene ontology annotations to assess functional similarity among orthologs and paralogs: A short report. PLoS Computational Biology, 8(2), e1002386. 22359495 10.1371/journal.pcbi.1002386 PMC3280971

[R30] Turner H, Bailey T, & Krzanowski W (2005). Improved biclustering of microarray data demonstrated through systematic performance tests. Computational Statistics & Data Analysis, 48, 235–254.

[R31] Whisenant TC, & Nigam SK (2022). Organic anion transporters (OAT) and other SLC22 transporters in progression of renal cell carcinoma. Cancers, 14(19), 4772. 36230695 10.3390/cancers14194772 PMC9563088

[R32] Wikipedia Contributors. (2018). Gene co-expression network-Wikipedia, the free encyclopedia. [Online; accessed February 6, 2019].

[R33] Xue Y, Liao Z, Li M, Luo J, Hu X, Luo G, & Chen W (2014). A new biclustering algorithm for time-series gene expression data analysis. In 2014 Tenth International Conference on Computational Intelligence and Security (pp. 268–272).

[R34] Zhang J (2010). A Bayesian model for biclustering with applications. Journal of the Royal Statistical Society, 59, 635–656.

