# Supplementary figures and images for: A Bayesian hierarchical hidden Markov model for clustering and gene selection: Application to kidney cancer gene expression data
Source: Biom J. Author manuscript; Available in PMC 2024 Jul 12. (PMC11239327; doi:10.1002/bimj.202300173)

(6) HMMBi-C

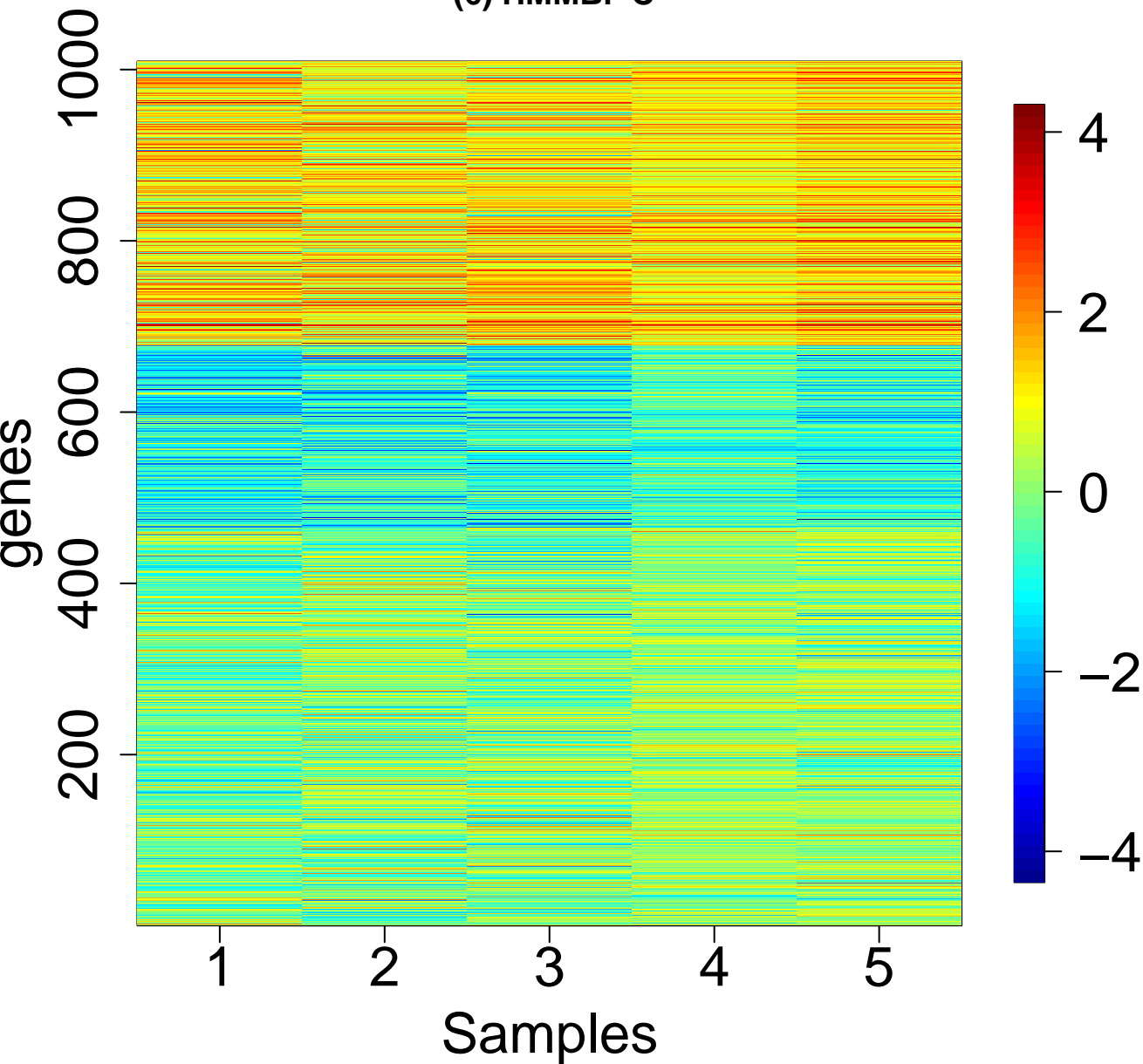

Supplement: Supporting information [file NIHMS2001776-supplement-Supporting_information.zip › Code_and_Data/FinalResults/Figure5ImageDataOrderedCluster6HMMBi-C.pdf]

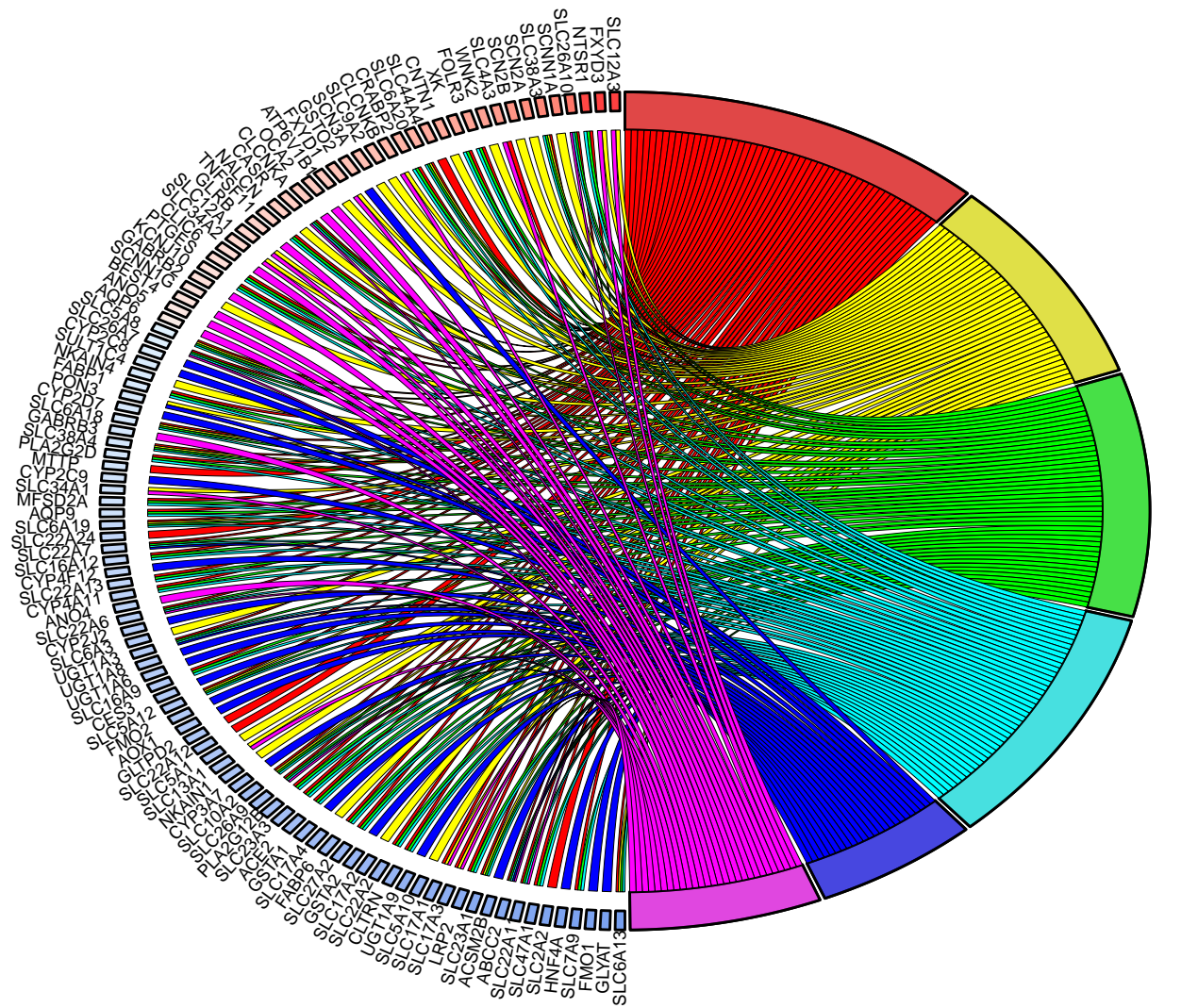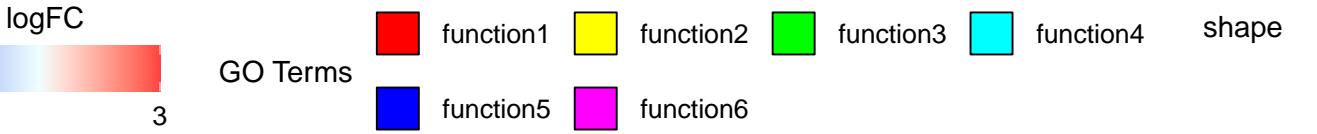

Supplement: Supporting information [file NIHMS2001776-supplement-Supporting_information.zip › Code_and_Data/FinalResults/Figure7Circular1HMMBi-C.pdf]

(3) HMMBi-C

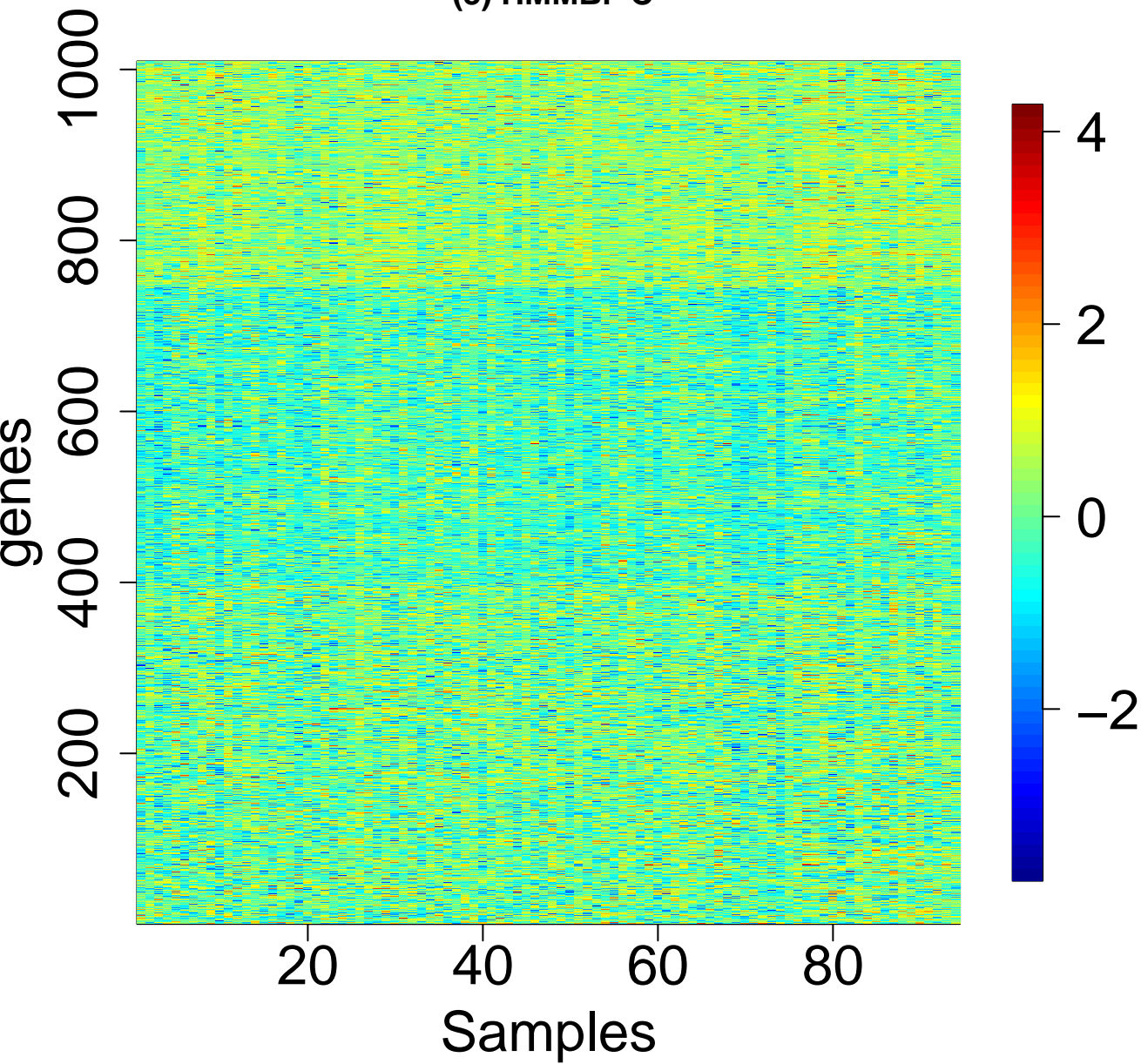

Supplement: Supporting information [file NIHMS2001776-supplement-Supporting_information.zip › Code_and_Data/FinalResults/Figure5ImageDataOrderedCluster3HMMBi-C.pdf]

(4) HMMBi-C

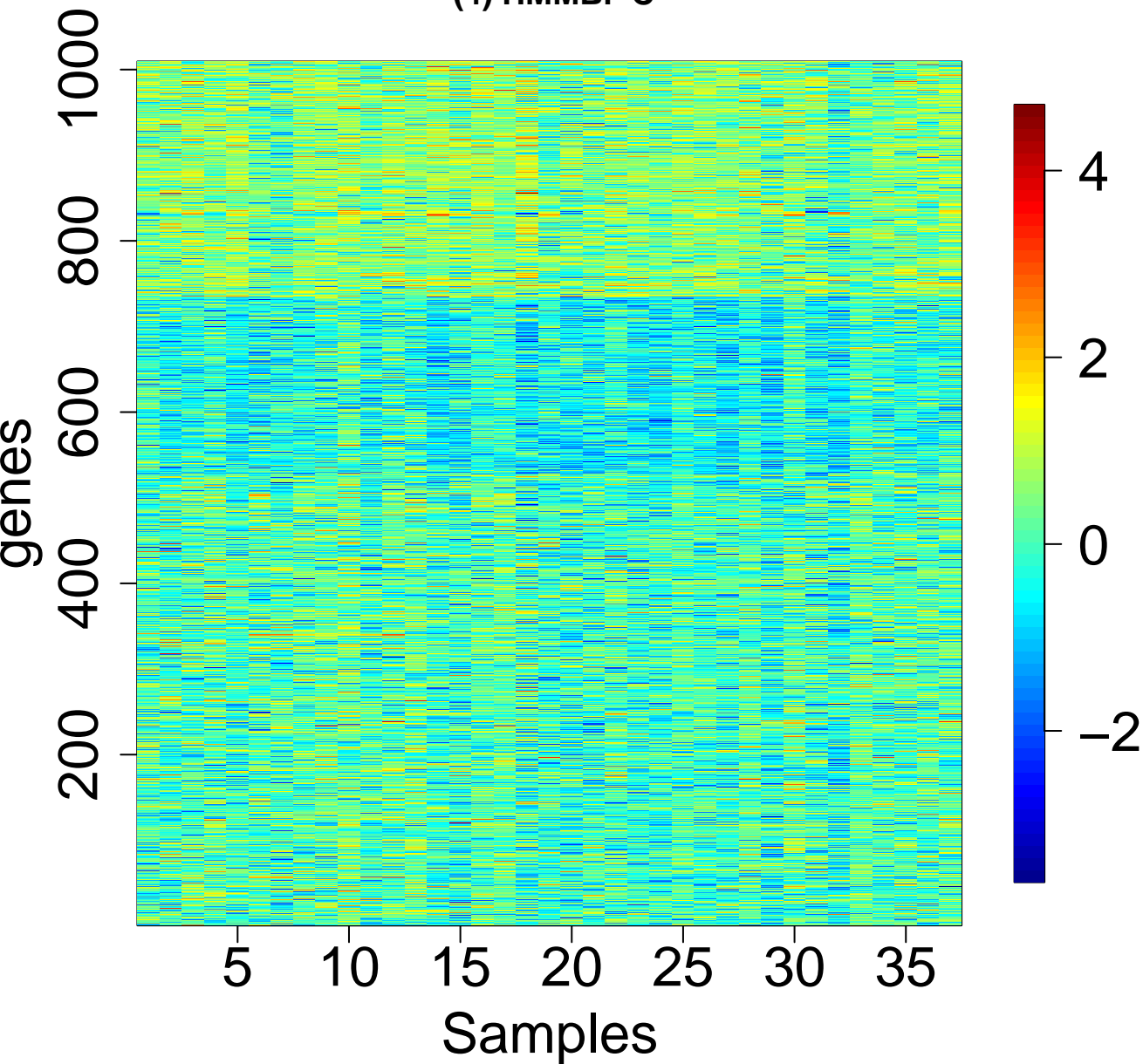

Supplement: Supporting information [file NIHMS2001776-supplement-Supporting_information.zip › Code_and_Data/FinalResults/Figure5ImageDataOrderedCluster4HMMBi-C.pdf]

(4) NoHMMBi-NoC

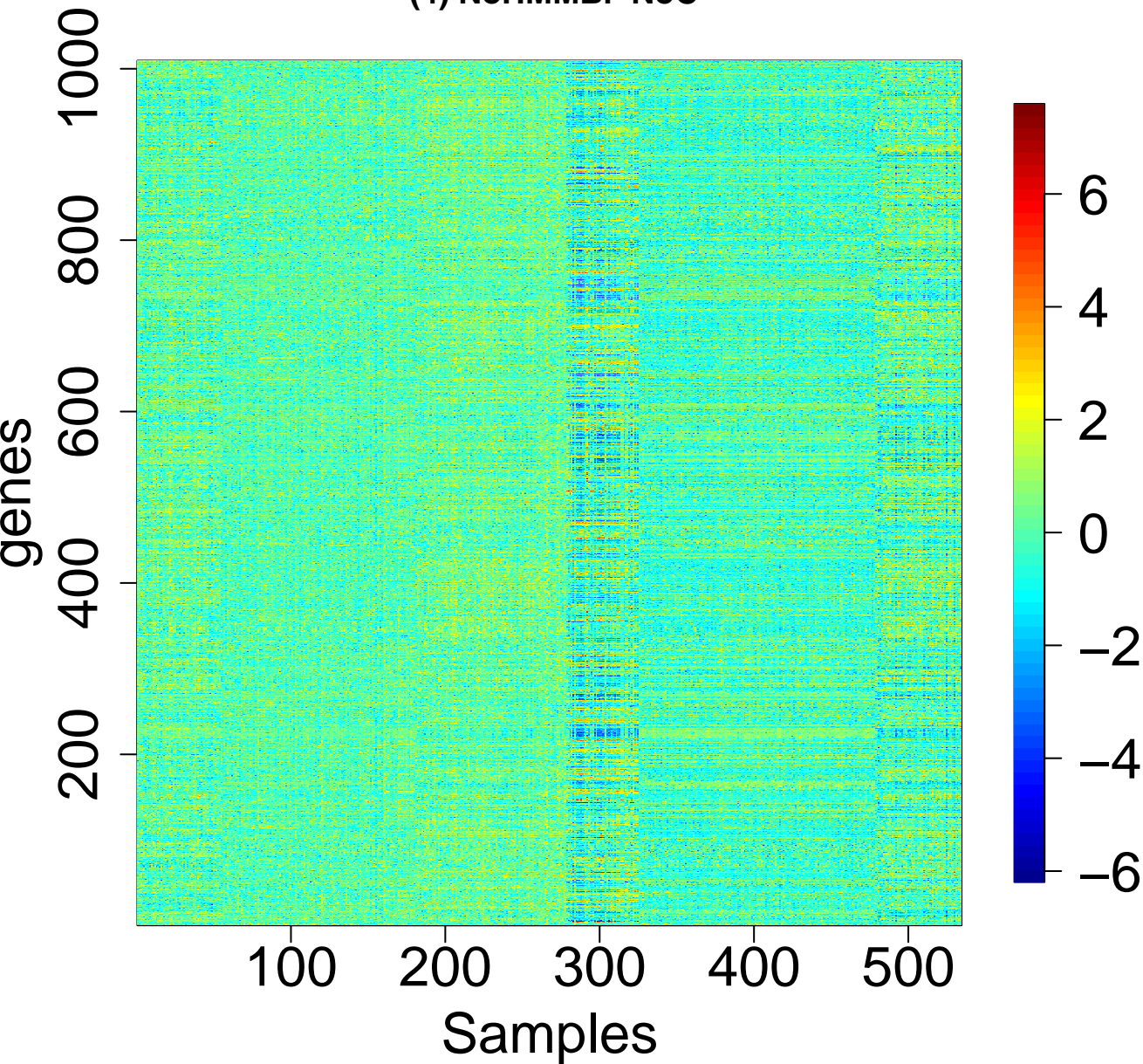

Supplement: Supporting information [file NIHMS2001776-supplement-Supporting_information.zip › Code_and_Data/FinalResults/Figure4ImageDataOrderedNoHMMBi-NoC.pdf]

**K = 8**

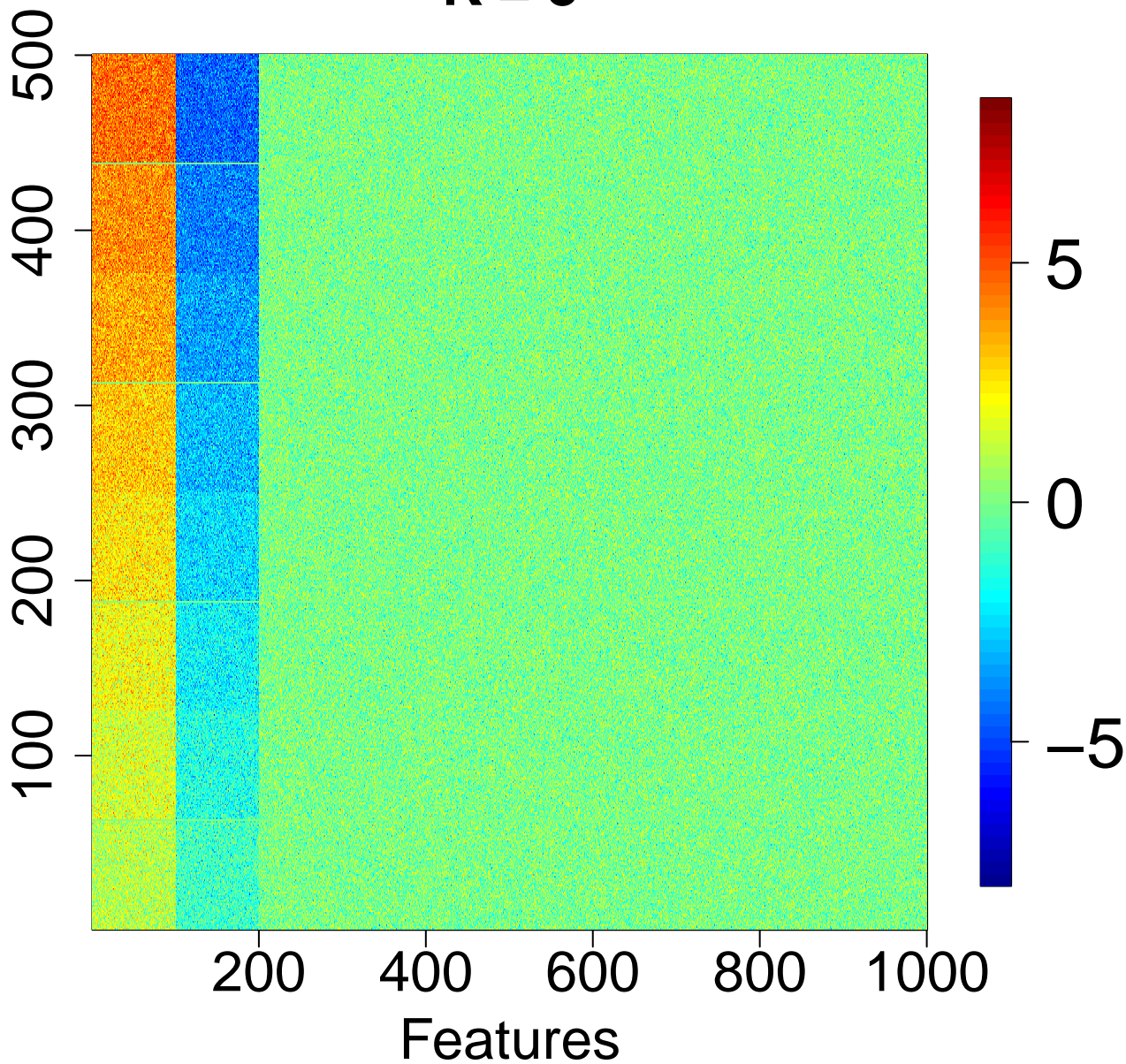

Supplement: Supporting information [file NIHMS2001776-supplement-Supporting_information.zip › Code_and_Data/FinalResults/Figure2Data8.pdf]

(1) HMMBi-C

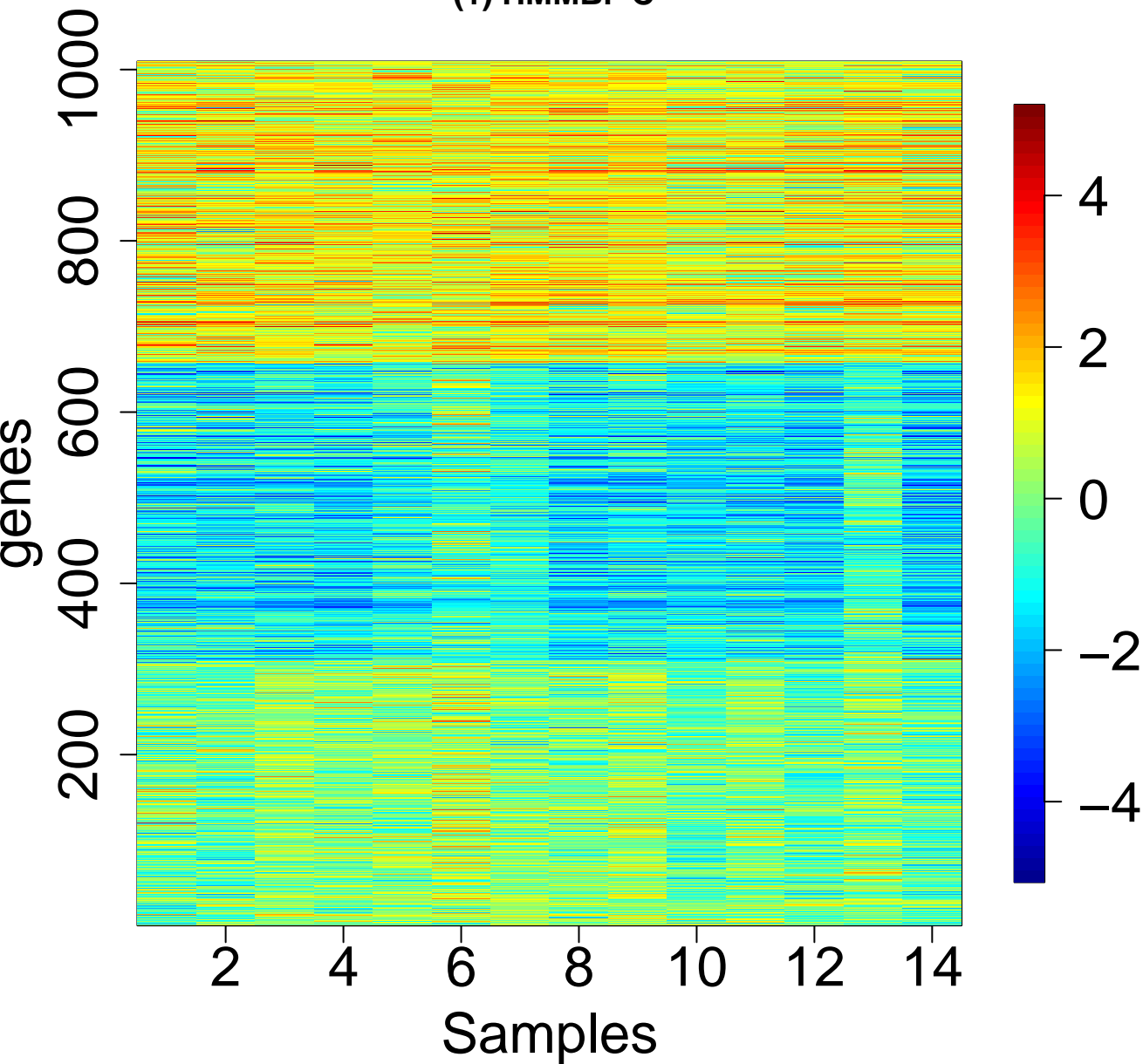

Supplement: Supporting information [file NIHMS2001776-supplement-Supporting_information.zip › Code_and_Data/FinalResults/Figure5ImageDataOrderedCluster1HMMBi-C.pdf]

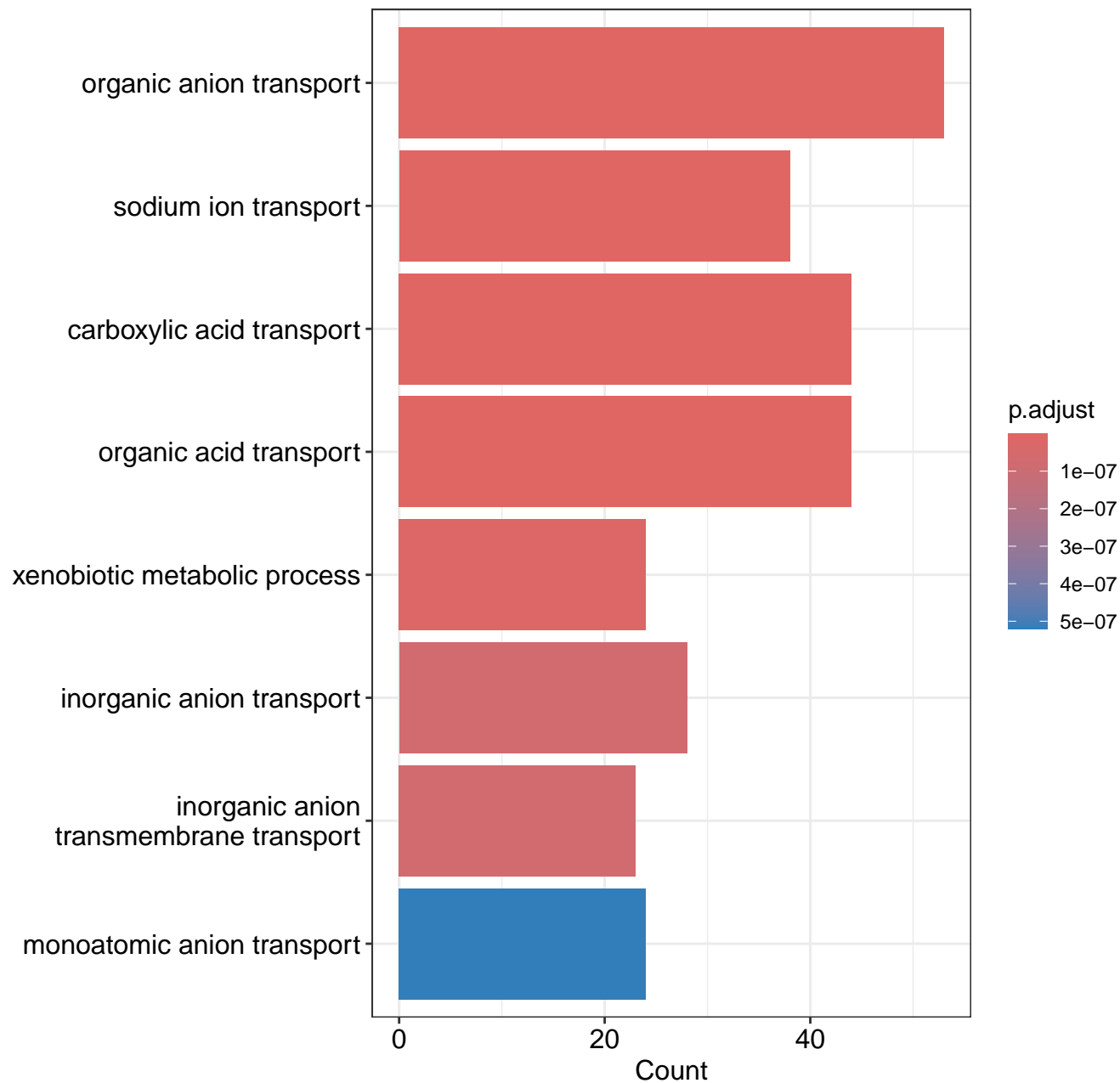

Supplement: Supporting information [file NIHMS2001776-supplement-Supporting_information.zip › Code_and_Data/FinalResults/Figure6BarPlot1HMMBi-C.pdf]

(11)

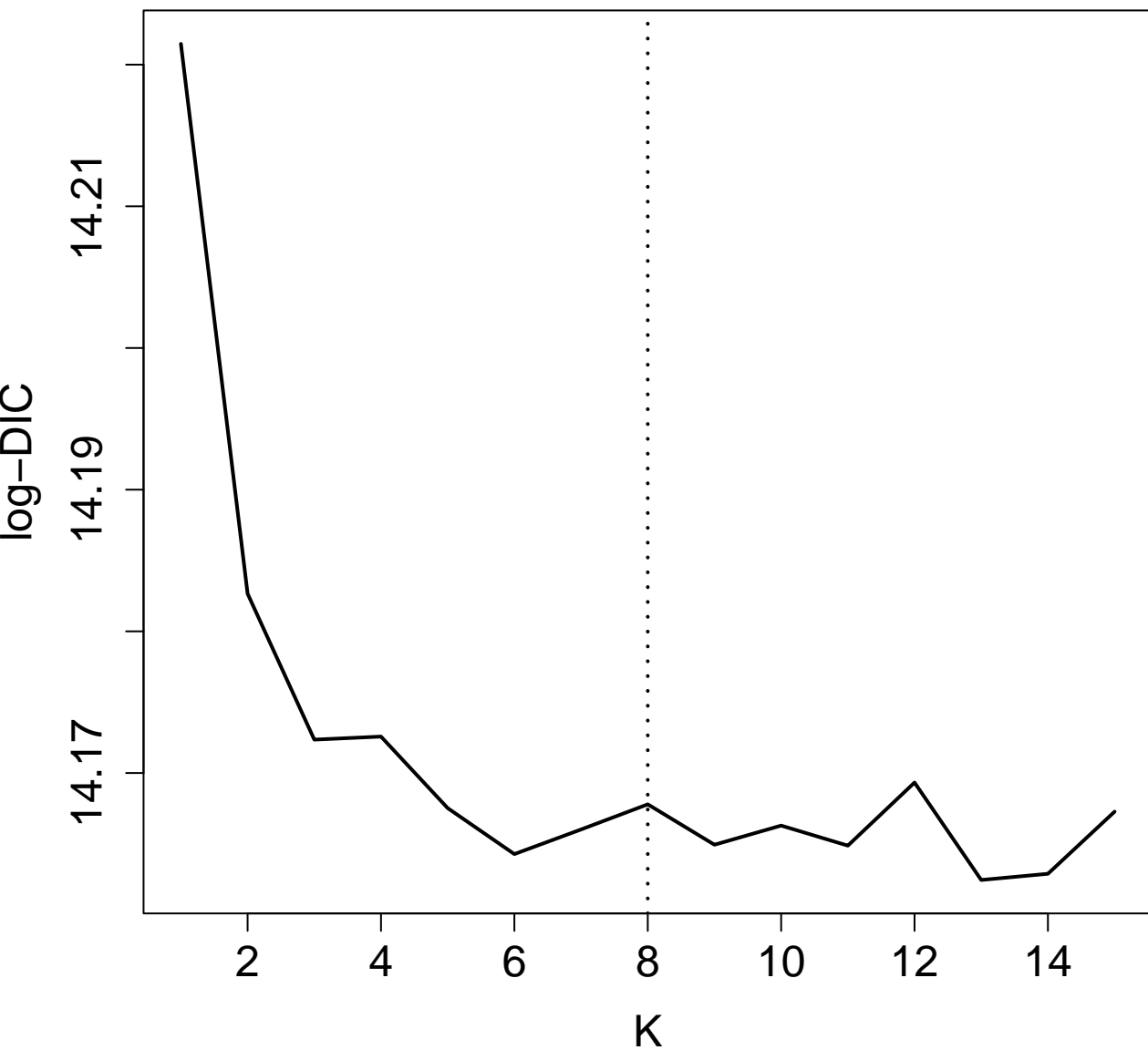

Supplement: Supporting information [file NIHMS2001776-supplement-Supporting_information.zip › Code_and_Data/FinalResults/Figure3DIC6.pdf]

(9)

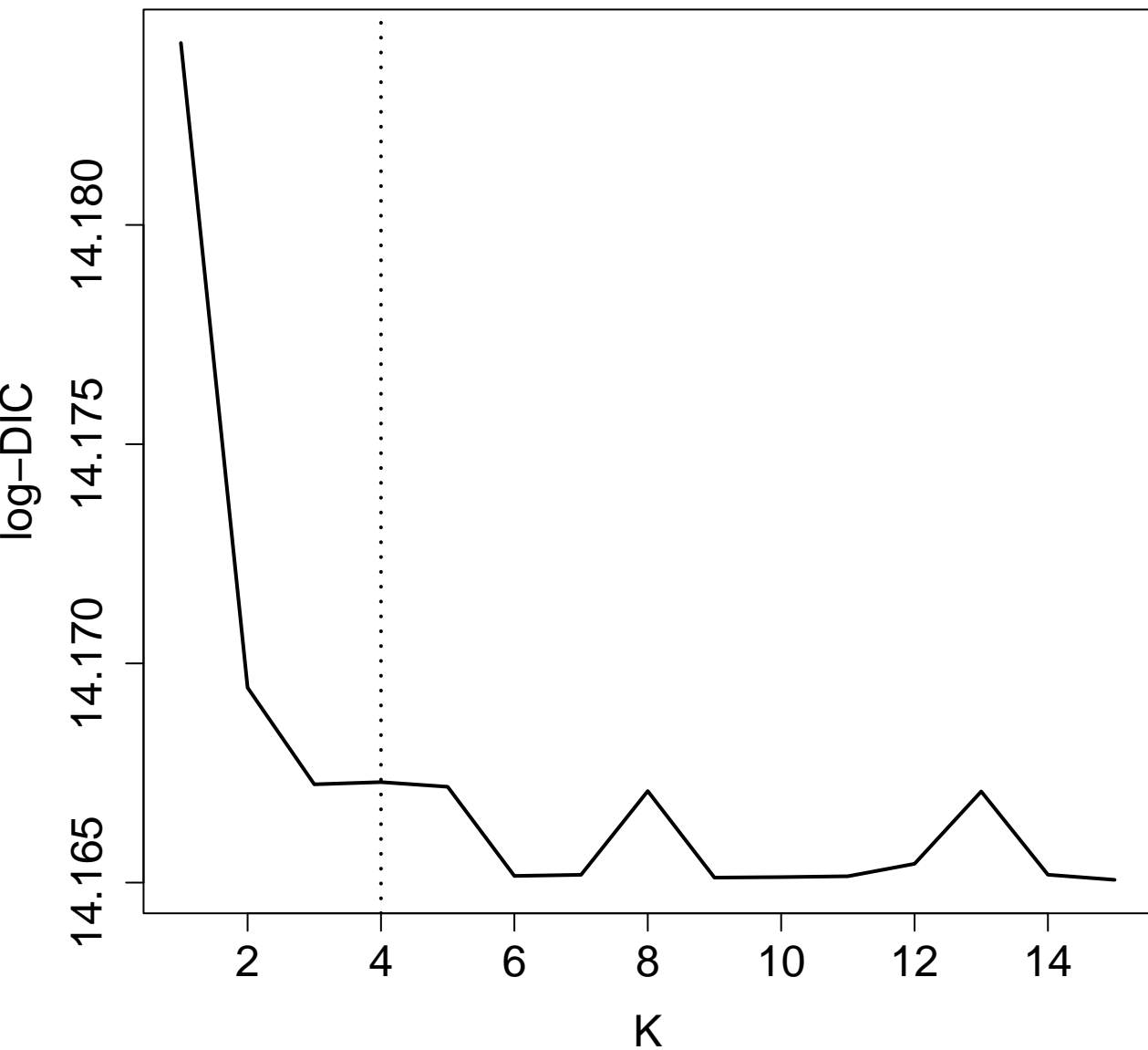

Supplement: Supporting information [file NIHMS2001776-supplement-Supporting_information.zip › Code_and_Data/FinalResults/Figure3DIC5.pdf]

(1) HMMBi-C

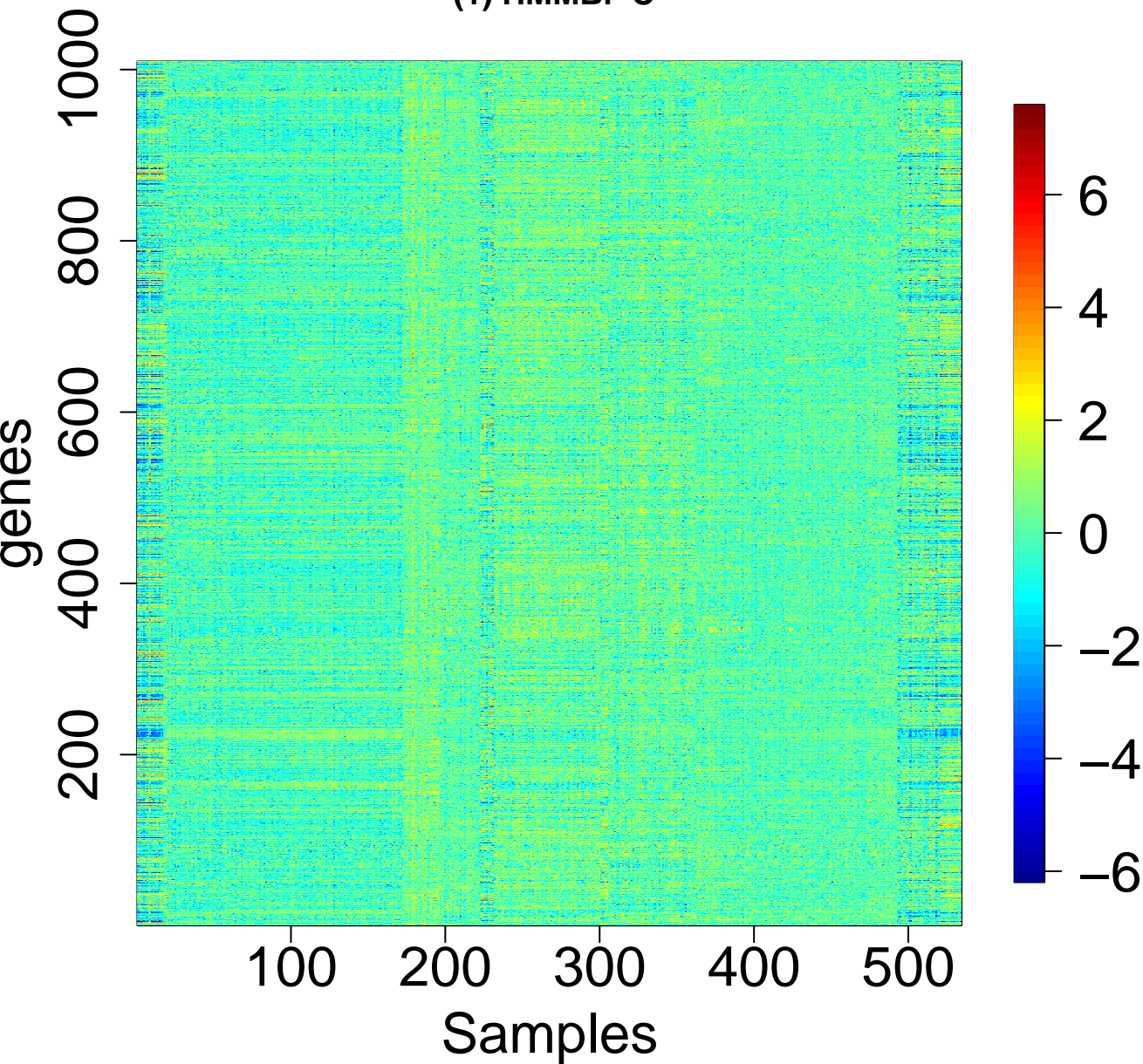

Supplement: Supporting information [file NIHMS2001776-supplement-Supporting_information.zip › Code_and_Data/FinalResults/Figure4ImageDataOrderedHMMBi-C.pdf]

(3) NoHMMBi-C

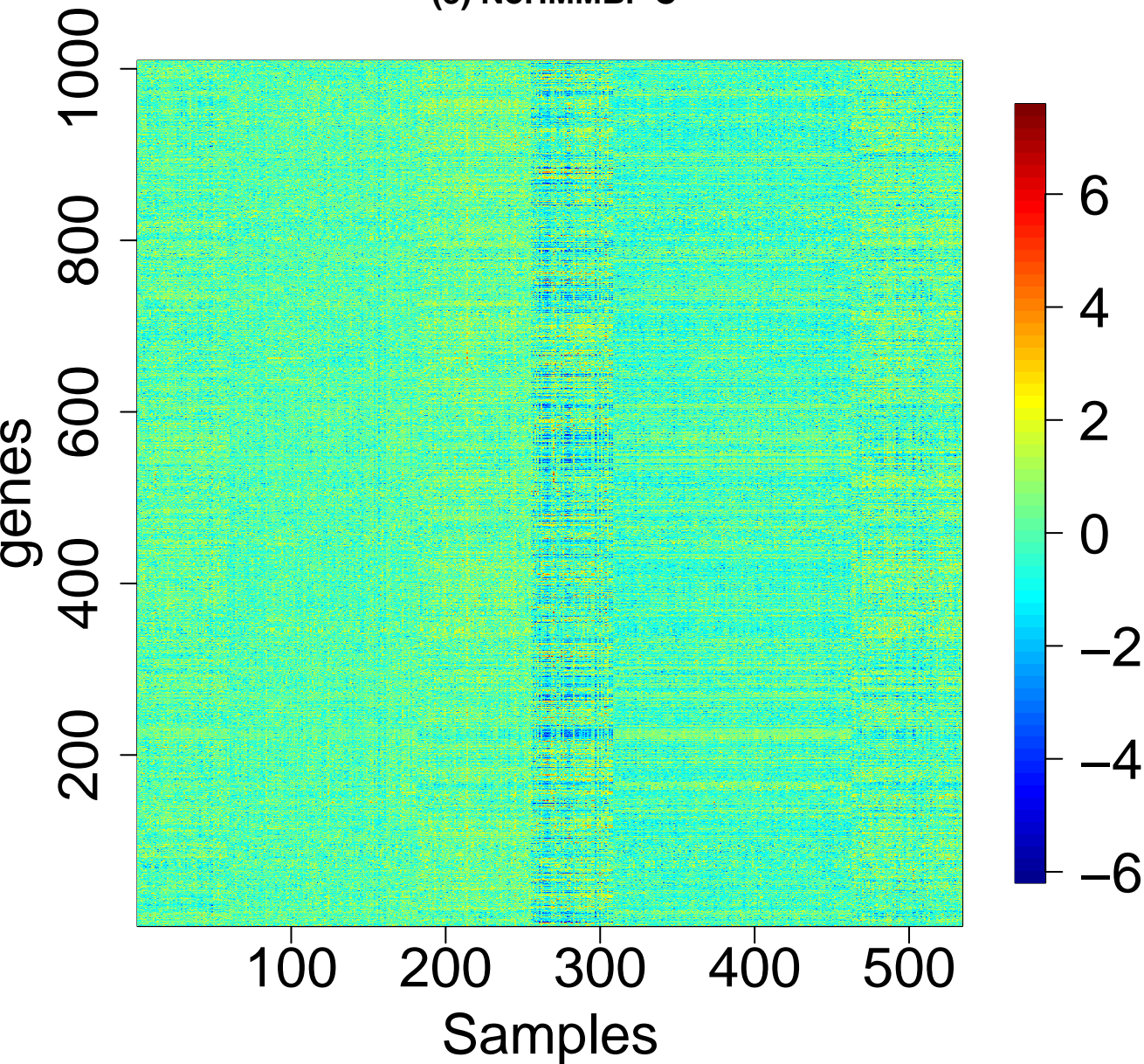

Supplement: Supporting information [file NIHMS2001776-supplement-Supporting_information.zip › Code_and_Data/FinalResults/Figure4ImageDataOrderedNoHMMBi-C.pdf]

(2) HMMBi-C

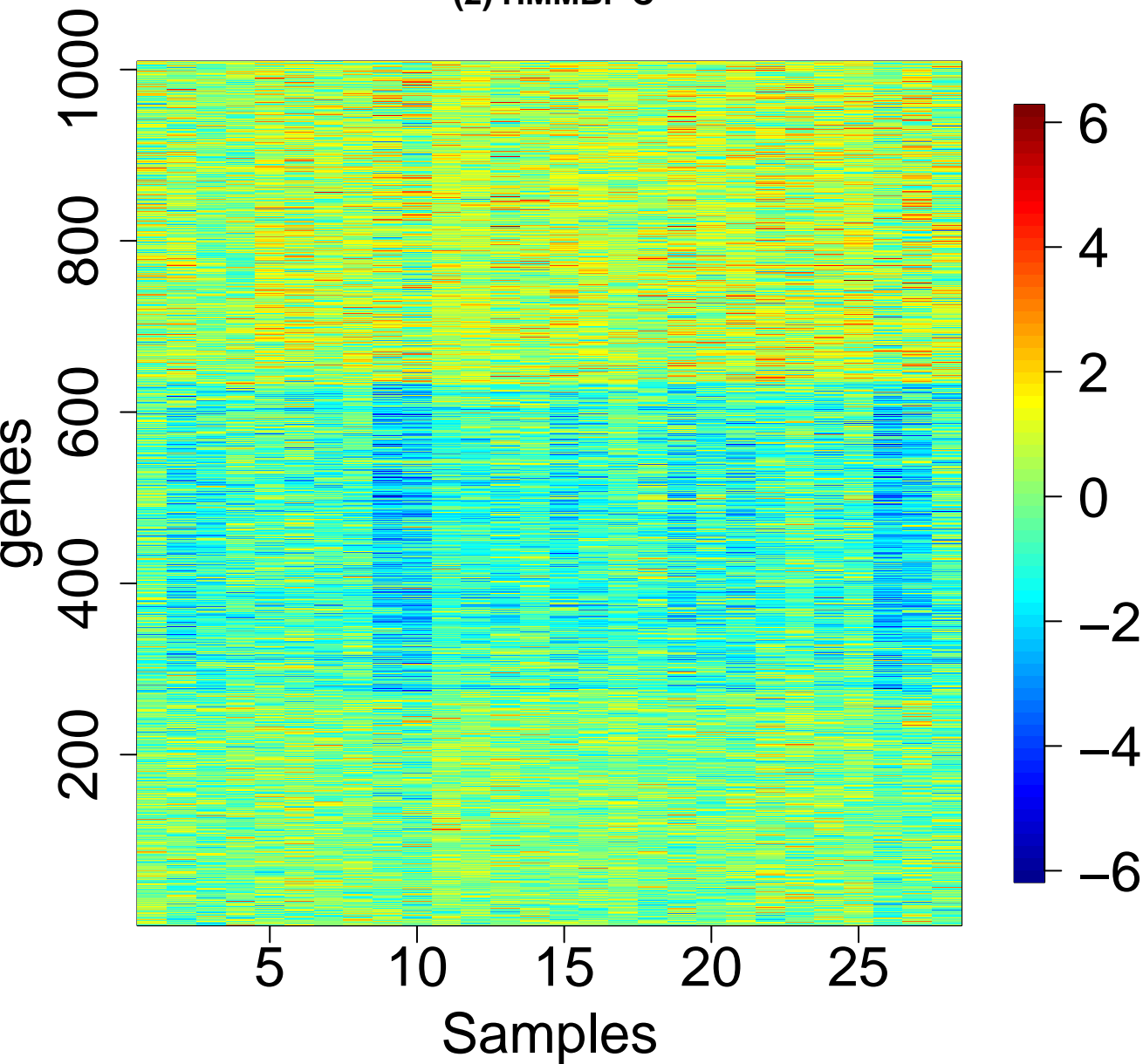

Supplement: Supporting information [file NIHMS2001776-supplement-Supporting_information.zip › Code_and_Data/FinalResults/Figure5ImageDataOrderedCluster2HMMBi-C.pdf]

(7)

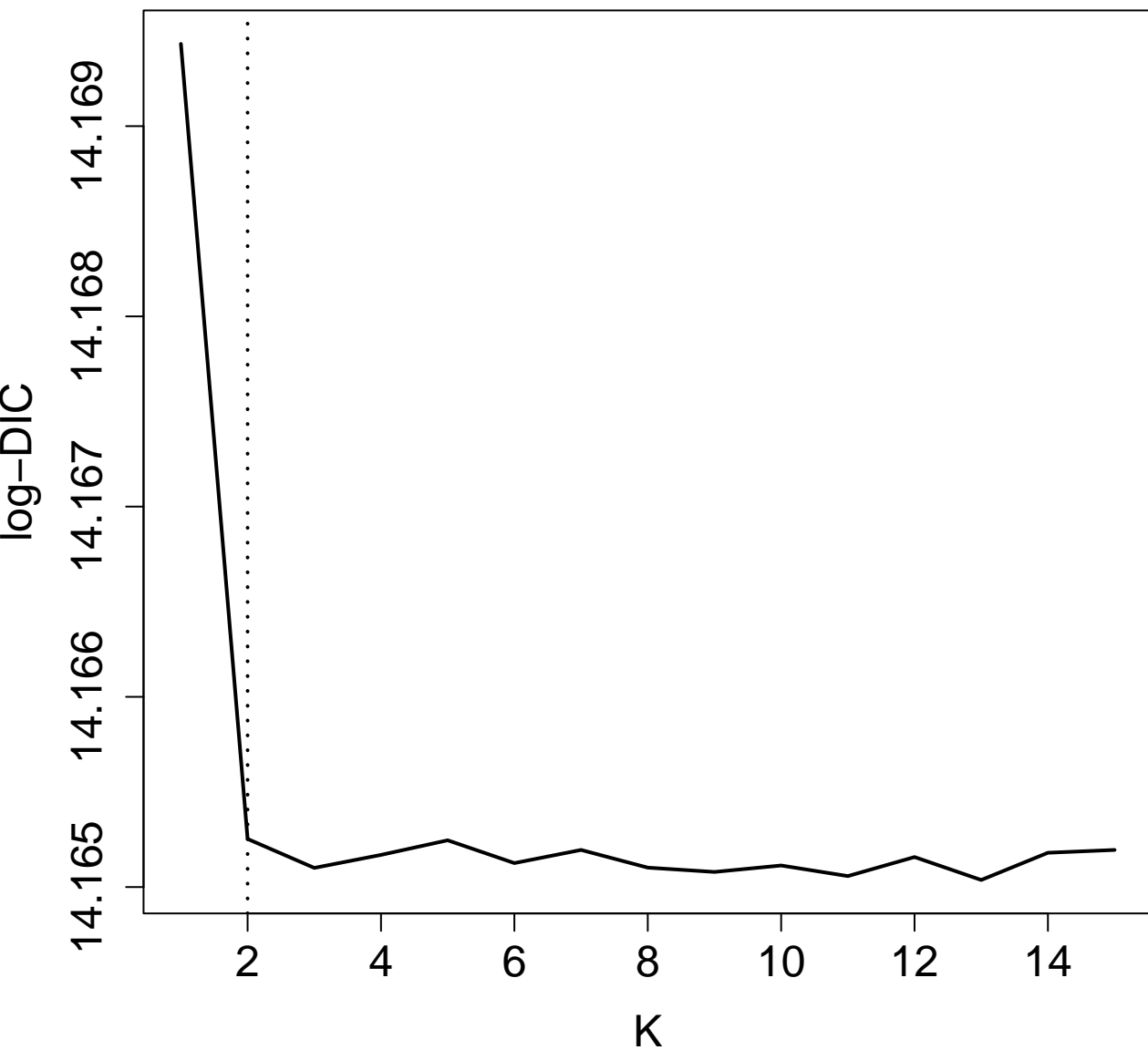

Supplement: Supporting information [file NIHMS2001776-supplement-Supporting_information.zip › Code_and_Data/FinalResults/Figure3DIC4.pdf]

**K = 4**

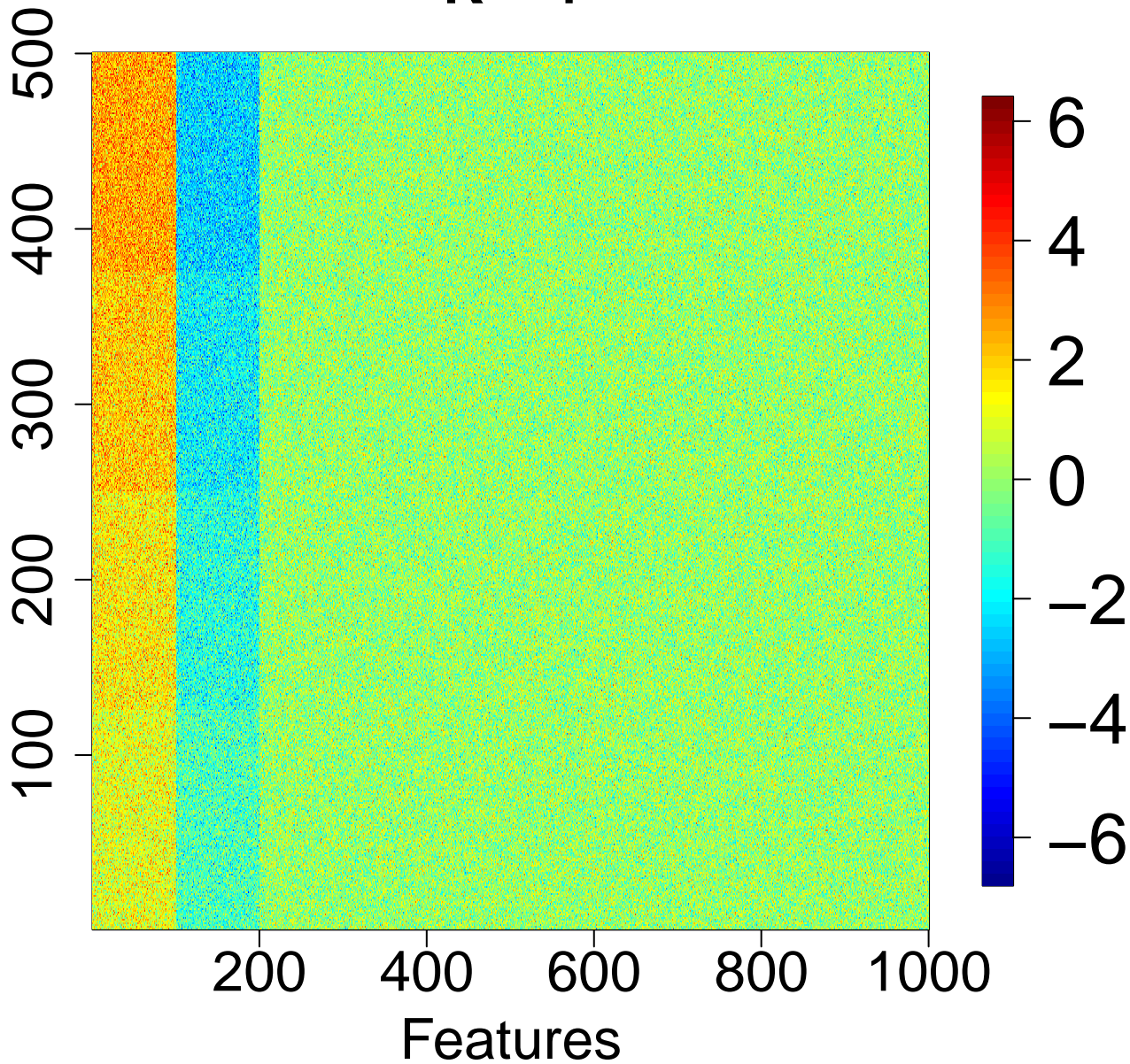

Supplement: Supporting information [file NIHMS2001776-supplement-Supporting_information.zip › Code_and_Data/FinalResults/Figure2Data4.pdf]

(1)

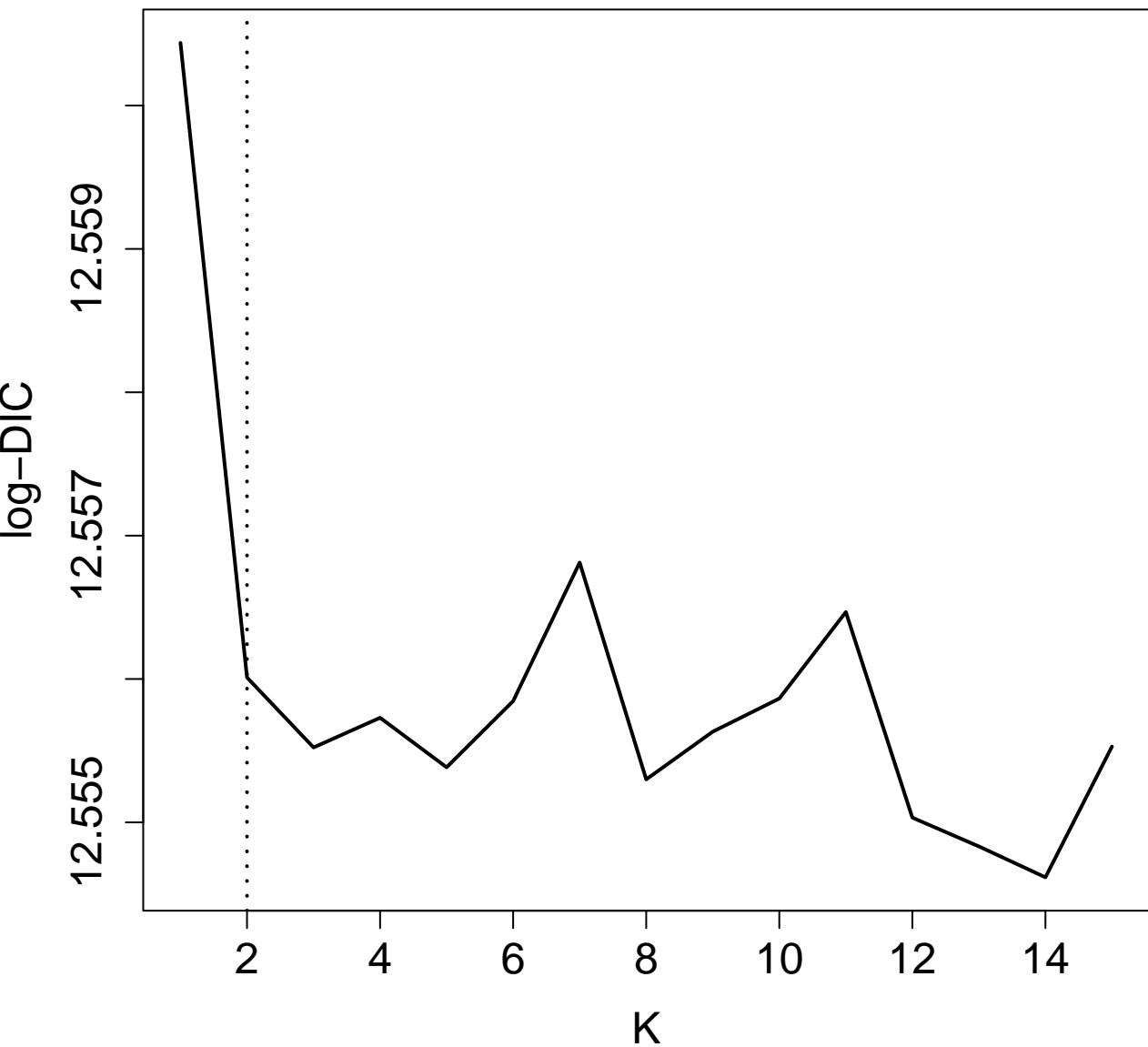

Supplement: Supporting information [file NIHMS2001776-supplement-Supporting_information.zip › Code_and_Data/FinalResults/Figure3DIC1.pdf]

(5)

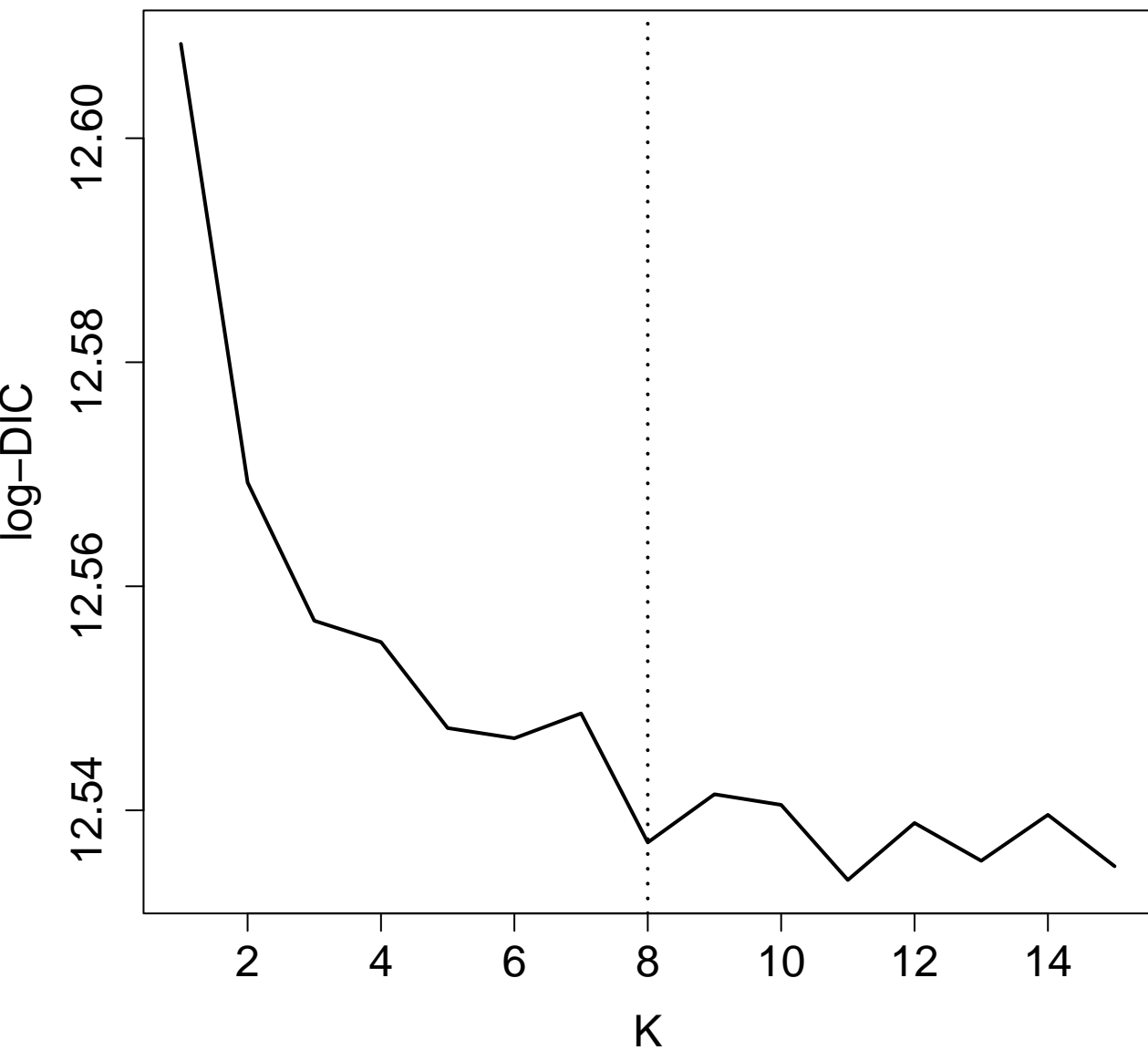

Supplement: Supporting information [file NIHMS2001776-supplement-Supporting_information.zip › Code_and_Data/FinalResults/Figure3DIC3.pdf]

(3)

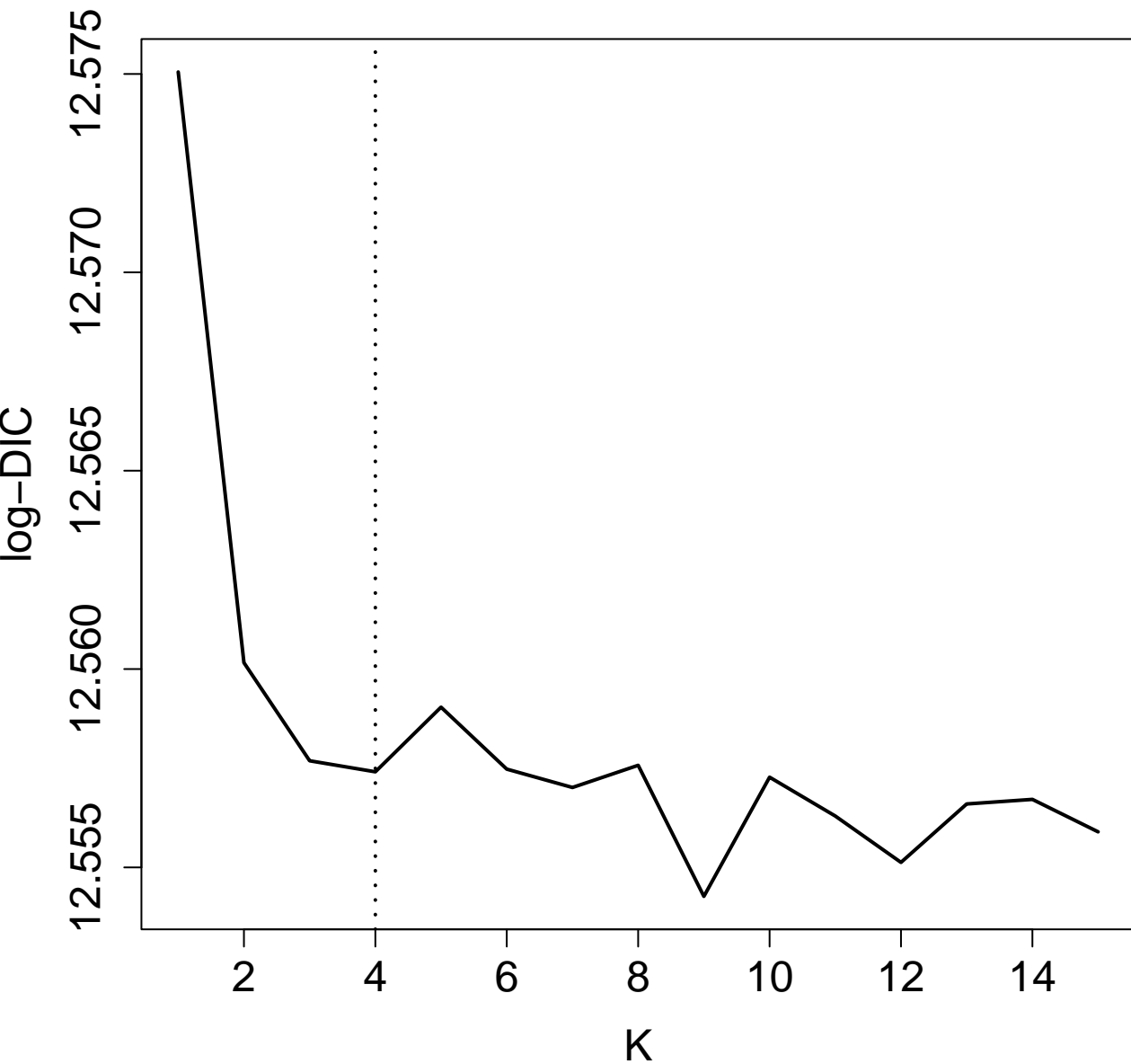

Supplement: Supporting information [file NIHMS2001776-supplement-Supporting_information.zip › Code_and_Data/FinalResults/Figure3DIC2.pdf]

**K = 2**

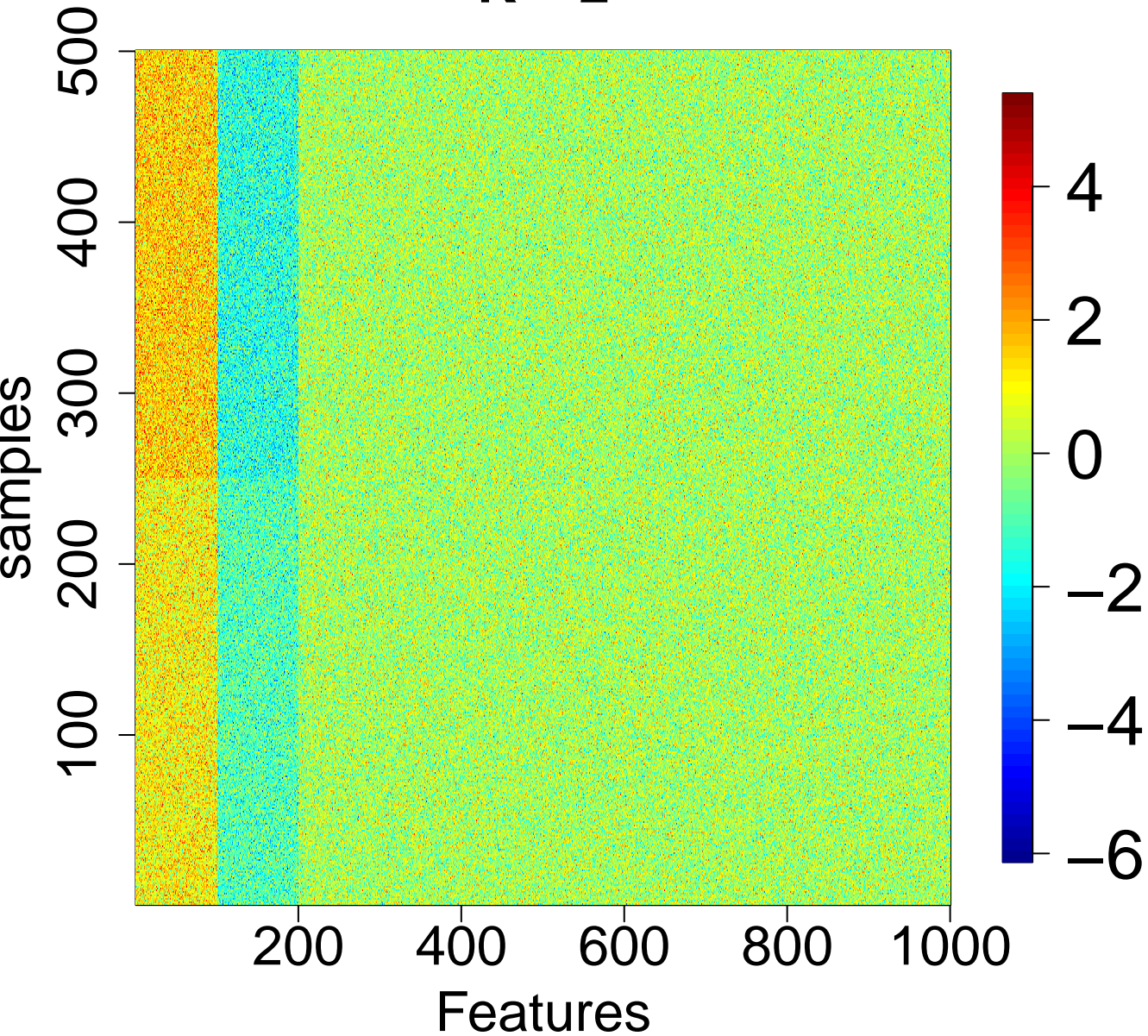

Supplement: Supporting information [file NIHMS2001776-supplement-Supporting_information.zip › Code_and_Data/FinalResults/Figure2Data2.pdf]

(5) HMMBi-C

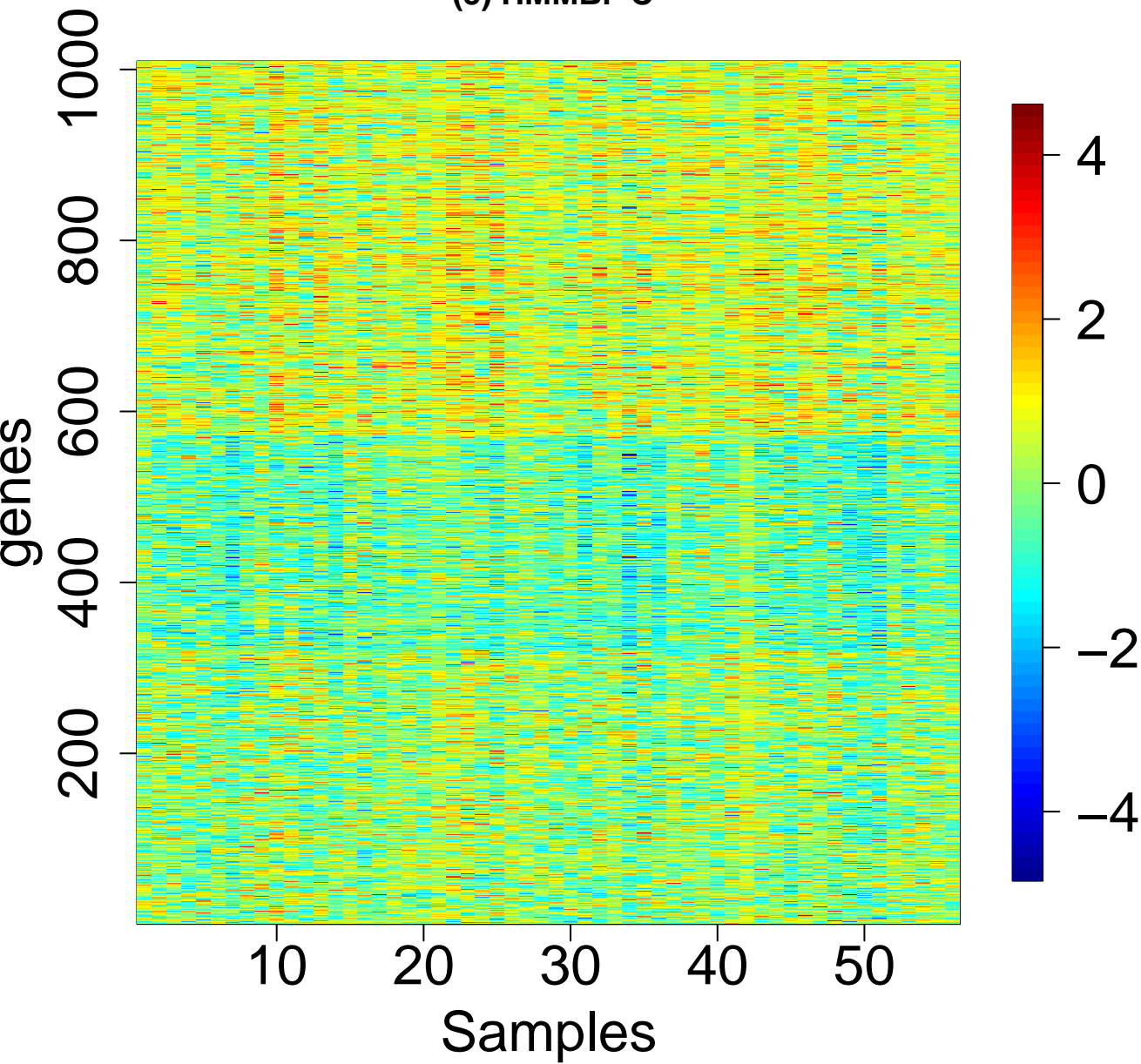

Supplement: Supporting information [file NIHMS2001776-supplement-Supporting_information.zip › Code_and_Data/FinalResults/Figure5ImageDataOrderedCluster5HMMBi-C.pdf]

(2) HMMBi-NoC

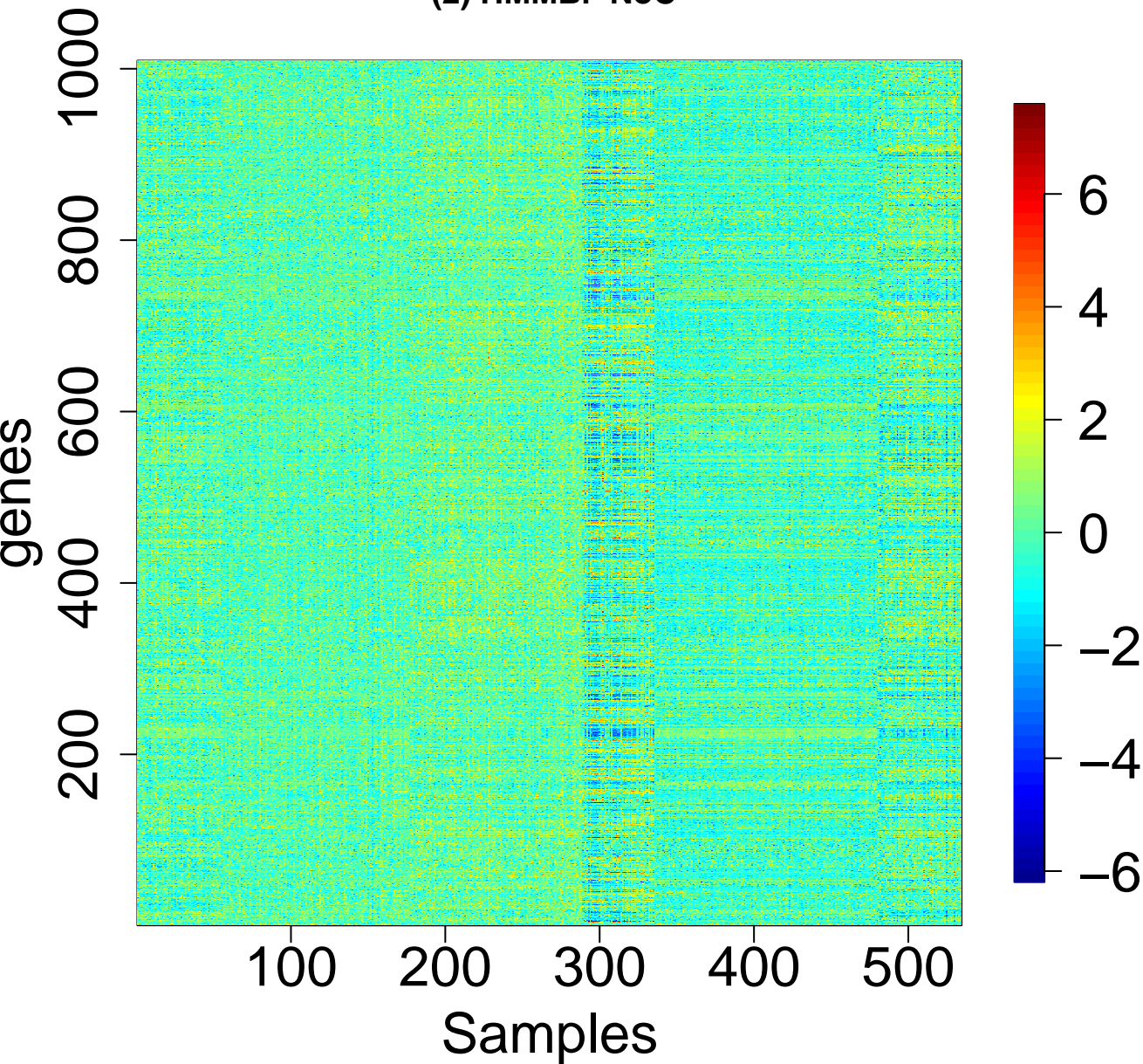

Supplement: Supporting information [file NIHMS2001776-supplement-Supporting_information.zip › Code_and_Data/FinalResults/Figure4ImageDataOrderedHMMBi-NoC.pdf]
